# Protein Solvent-Accessibility Prediction by a Stacked Deep Bidirectional Recurrent Neural Network

**DOI:** 10.3390/biom8020033

**Published:** 2018-05-25

**Authors:** Buzhong Zhang, Linqing Li, Qiang Lü

**Affiliations:** 1 School of Computer Science and Technology, Soochow University, Suzhou 215006, China; 20154027005@stu.suda.edu.cn (B.Z.); linqinglee@gmail.com (L.L.); 2 School of Computer and Information, Anqing Normal University, Anqing 246011, China

**Keywords:** solvent-accessibility prediction, bidirectional recurrent network, sequence profile, merging operator

## Abstract

Residue solvent accessibility is closely related to the spatial arrangement and packing of residues. Predicting the solvent accessibility of a protein is an important step to understand its structure and function. In this work, we present a deep learning method to predict residue solvent accessibility, which is based on a stacked deep bidirectional recurrent neural network applied to sequence profiles. To capture more long-range sequence information, a merging operator was proposed when bidirectional information from hidden nodes was merged for outputs. Three types of merging operators were used in our improved model, with a long short-term memory network performing as a hidden computing node. The trained database was constructed from 7361 proteins extracted from the PISCES server using a cut-off of 25% sequence identity. Sequence-derived features including position-specific scoring matrix, physical properties, physicochemical characteristics, conservation score and protein coding were used to represent a residue. Using this method, predictive values of continuous relative solvent-accessible area were obtained, and then, these values were transformed into binary states with predefined thresholds. Our experimental results showed that our deep learning method improved prediction quality relative to current methods, with mean absolute error and Pearson’s correlation coefficient values of 8.8% and 74.8%, respectively, on the CB502 dataset and 8.2% and 78%, respectively, on the Manesh215 dataset.

## 1. Introduction

Residue solvent accessibility (RSA) [1] in a protein is defined as the extent of accessible surface area of a given residue and is related to the residue spatial arrangement and packing. It reveals the folding state of proteins and has been considered as a significant quantitative measurement for three-dimensional structures of proteins [2]. Solvent accessibility is closely involved in structural domains’ identification [3], fold recognition [4], binding region identification [5], protein-protein interactions [6] and protein-ligand interactions [7]. Therefore, predicting the RSA of a protein represents an important step in determining its structure and function. Traditionally, RSA prediction is performed in two forms: (1) as a binary or multi-class classification problem with varying thresholds (two-state (exposed or buried) [8] or three-state (exposed, intermediate, or buried)) [9]; and (2) based on the relative accessible solvent area (rASA) prediction [10]. For example, if the surface area of a residue exceeds a threshold of 25%, the residue is classified as exposed; however, there is no standard definition of the thresholds for solvent-accessible area. Generally, the later approach is preferred over the former since rASA provides more information compared to binary or multi-class classification. For instance, it provides numerical values, which is required to apply this characteristic in protein structure and function prediction. In view of this, it is necessary to predict rASA. Recently, more and more studies have focused on rASA prediction.

Machine learning methods are extensively applied in RSA prediction and include those related to sequence alignment [11], neural networks [12,13,14,15], support vector machines [16,17], the use of multiple linear-regression models [18], Bayesian statistics [19], nearest-neighbor methods [20], energy optimization [21], gradient-boosted regression trees [22] and deep learning [23].

In 2003, Ahmad et al. [10] firstly detailed rASA prediction, and more attention has been paid to this research since that time. Ahmad et al. proposed a neural network method with only single sequence information as the input features, and the result of 18.8% mean absolute error (MAE) was achieved on the CB502 dataset. Wang et al. [18] applied the multiple linear regression method to predict rASA from the sequence information and position-specific scoring matrix (PSSM). This method achieved a result of 16.2% MAE on the CB502 dataset. Wang et al. [17] improved the result to 15.1% on the same dataset by accumulation cutoff set and support vector machine. Using a weighted sliding window scheme, Zhang et al. [24] obtained the result of 14% MAE on the CB502 dataset. Fan et al. [22] used gradient boosted regression trees to predict rASA and achieved a state-of-the-art performance, which is 9.4% MAE and 0.73 Pearson’s correlation coefficient (PCC) on the CB502 dataset. Another benchmark dataset Manesh215 [25] is also widely used by researchers [10,13,14,22,24,26] to validate prediction methods. Table 1 summarizes the recent developments in predicting the values of rASA.

Position-specific scoring matrix has been widely used in proteomics and bioinformatics, such as protein structure prediction [31,32], backbone angles [23],protein subcellular localization prediction [33,34,35], membrane protein type prediction [36,37,38], protein subchloroplast localization [39], etc. Protein-sequence coding and PSSM have also been used for RSA prediction [12,16,20]. Recently, other sequence-based features, such as physical properties [40], conservation score [41] and side-chain environment [42], have been used [20,21,22,23]. Additionally, properties predicted by computational methods have also been used, including those associated with evaluating secondary structure and protein disorder [21,22,43]. For most of these, standard labels for RSA areas are calculated by DSSP software [44]. The rASA is obtained by normalizing the ASA value over the maximum value of exposed surface area obtained for either (1) each amino acid or for (2) an extended tripeptide conformation of Ala-X-Ala or Gly-X-Gly [10].

Although these methods for RSA prediction have progressed, RSA prediction remains challenging. Improved performance in these areas will enable more precision in related protein studies.

Recently, deep learning methods have dramatically improved the state-of-the-art in speech recognition, visual object recognition, object detection and many other domains [45]. The Recurrent Neural Network (RNN), a type of deep neural network, processes an input sequence one element at a time, maintaining a hidden vector that implicitly contains the history information about the past elements of the sequence. RNN is an extremely powerful sequence model for sequence modeling tasks [46], and many RNN methods have been applied to protein structure and function prediction [47,48,49]. We focused on a RNN model specific to protein sequences and applied a bidirectional recurrent neural network (BRNN) to predict RSA. To capture more local and long-range information, a merging operator was used to merge bidirectional information, given that this has rarely been addressed previously. Our deep learning method, a stacked deep bidirectional recurrent neural network (SDBRNN), is proposed, with three-layer bidirectional long-short memory (BLSTM) network with different merging operators used in each hidden layer. The public benchmark datasets CB502, Manesh215 and CASP10 were used for testing, with our experimental results showing that SDBRNN outperformed current state-of-the-art methods. Our method represents a more general approach and can be applied to a broad range of problems within and outside of computational biology. The rASA prediction tool of SDBRNN, training and testing datasets can be download from http://210.45.175.81:8080/rsa/sdbrnn.html.

## 2. Results

### 2.1. Measurement Evaluation

To evaluate model performance for RSA prediction, four widely-used measures were used: MAE, PCC, accuracy (ACC) and Matthews’ correlation coefficient (MCC). The formulas are defined in Equations (1)–(4).

(1) $$\begin{array}{c} MAE=\frac{\sum_{i=1}^{N}\left|RSAr_{i}-RSAp_{i}\right|}{N} \end{array}$$

(2) $$\begin{array}{c} PCC=\frac{\sum_{i=1}^{N}\left(RSAr_{i}-\bar{RSAr}\right)\left(RSAp_{i}-\bar{RSAp}\right)}{\sqrt{\left[\sum_{i=1}^{N}{\left(RSAr_{i}-\bar{RSAr}\right)}^{2}\right]\left[\sum_{i=1}^{N}{\left(RSAp_{i}-\bar{RSAp}\right)}^{2}\right]}} \end{array}$$

In Equations (1) and (2), $RSAr_{i}$ and $RSAp_{i}$ are the real and predicted rASA values of the *i*-th residue in the sequence, respectively, while $\bar{RSAr}$ and $\bar{RSAp}$ are the corresponding mean values. *N* is the length of the protein sequence to predict. MAE is used to evaluate the deviation between the predicted and real relative values of RSA, and PCC is employed to quantify the relationship between predicted and real values.

(3) $$\begin{array}{c} ACC=(TP+TN)/N \end{array}$$

(4) $$\begin{array}{c} MCC=\frac{TP\times TN-FP\times FN}{\sqrt{\left(TP+FP)(TP+FN)(TN+FP)(TN+FN\right)}} \end{array}$$

MAE and PCC were commonly used to measure continuous values, whereas ACC and MCC were often used to measure binary classification. Prediction accuracy is the true predicted residues divided by the length of predicted sequence. The MCC is a correlation coefficient between the observed and predicted binary classifications. MCC is able to recover the drawback of accuracy regarding unbalance data. In Equations (3)–(4), TP,FP,TN and FN represent the number of true positives (exposed residues), true negatives (buried residues), false positives and false negatives, respectively.

### 2.2. Performance on Relative Solvent-Accessible Area Prediction

**Figure 1:**
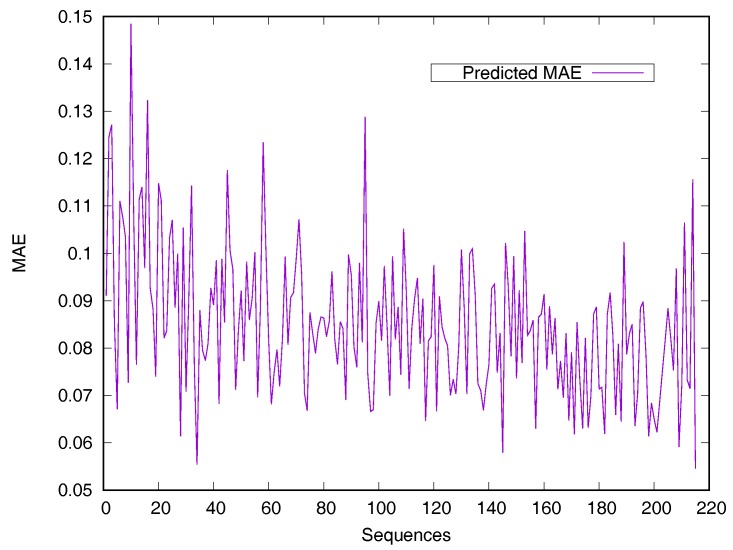
Predicted MAE based-on individual sequence from the Manesh215 dataset. The protein sequences are ordered by sequence length.

RSA predictors that use machine learning methods are converted into regressive problems. Results from the SDBRNN on the CB502 and Manesh215 datasets are shown in Table 2. Five predictors, including SARpred [13], SVR [13], Real-SPINE [15], NetSurfP [50] and PredRSA [22], the results of which are state-of-the-art, were used for comparison. Our method achieved an MAE of 8.8% and a PCC of 75% on CB502, an MAE of 8.2% and a PCC of 78% on Manesh215 respectively, both of which are better than the state-of-the-art. Additionally, PCC values for the CB502 and Manesh215 datasets were 2% and 3% higher than that of PredRSA. To evaluate the generalization on individual sequences, the predicted MAEs of individual sequences from the Manesh215 dataset have been further analyzed in Figure 1. The x-axis represents the sequence length. The MAE values were mostly from 0.065–0.1. In general, prediction performances on long sequences are higher than those of short sequences.

Two publicly-available datasets (CASP10 and CASP11) were also used to validate SDBRNN. CASP10 contains 90 proteins, and CASP11 contains 72 proteins, with the lengths of all sequences between 50 and 600 residues. The PCC value for the CASP10 dataset was 74.2%, which was 3.2% better than that for PredRSA, and that for CASP11 was 74.3%, which was slightly better than SPIDER2 [23].

Another representative method (PSO-SVR [24]) was also compared. The maximum solvent-accessible area standard used in that study was according to Adhamd’s work [10], which used extended tripeptides (Ala-X-Ala). We re-prepared data using the standard and re-trained model. Experiments using our method showed MAE and PCC values of 12.0%, and 0.765, respectively, on the Manesh215 dataset, which were better than 13.2% and 0.74 achieved by PSO-SVR. Additionally, on the CB502 dataset, our method showed MAE and PCC values of 13.3% and 0.739, which were better than 14% and 0.73 obtained by PSO-SVR.

### 2.3. Comparison of Different Classification Predictors

There are many methods proposed for predicting binary classification (exposed or buried) of residues. Thus, we have also examined the performance of our method in terms of two-state predictions. Similar to the two-layer predictor strategy [37,38], firstly the predicted rASA values were generated by the SDBRNN model. Then, the predicted relative values were transformed into binary states with predefined thresholds for comparison. For instance, the residue with a rASA value that is greater than or equal to the threshold, it can be considered as exposed, otherwise, it is considered as buried. The predictive ability between methods was compared against SARpred [13], PR [29], SVR [28], two-stage SVR [27], SS-SVM [30] and PredRSA using the Manesh215 dataset. Table 3 shows the performances of different methods. Results indicated that the SDBRNN model performed better at RSA prediction and generalization, except for the threshold of 50%.

**Figure 2:**
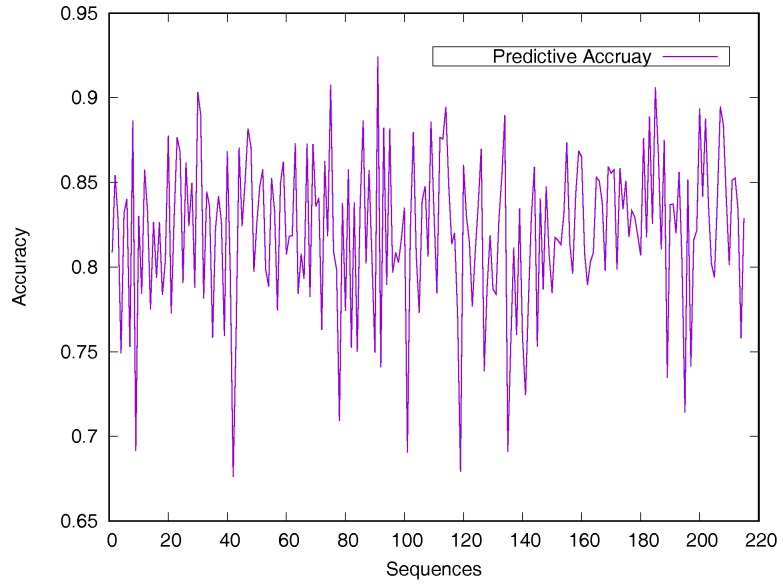
Predicted accuracy on individual sequences from the Manesh215 dataset. The rASA threshold is 25%. Protein sequences are ordered by sequence length.

Predicted accuracy on individual sequence from the Manesh215 dataset is analyzed in Figure 2 for evaluating the model generalization. The prediction accuracy of most proteins is between 75% and 90%. Only 14 sequences out of the 215 proteins have a prediction accuracy of less than 75%.

We also tested our model using three publicly-available datasets at different thresholds: CB502, Manesh215 and CASP10. ACC and MCC results are listed in Table 4 and Table 5. To compare with PredRSA [22] in detail, its results are also listed in the tables. Our method showed mostly better accuracy on CB502 and Manesh215, except the threshold of 50%. At a threshold of 50%, the ACC associated with the PredRSA on the CB502 and Manesh215 datasets was 0.6% and 0.2% better than our method, respectively. The MCC results of our method are still better than PredRSA.

### 2.4. Residue-Specific Variation in Predictive Error

**Figure 3:**
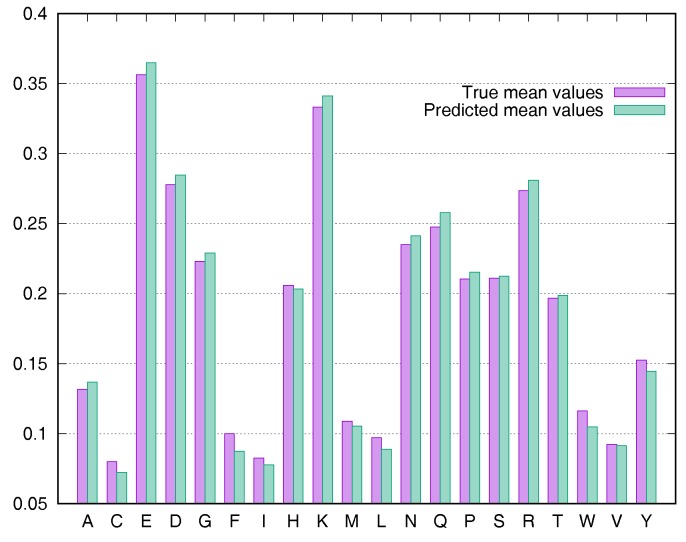
Comparison of true mean values and predicted mean values for 20 types of amino acids using the CB502 dataset.

To evaluate the predictive performance for various types of residues, we calculated the average rASA values using the Manesh215 and CB502 datasets for all 20 types of amino acids. SDBRNN accurately predicted RSA using the CB502 dataset with an MAE <1%, except that phenylalanine (F) and tryptophan (W) were predicted with an MAE <1.3% (Figure 3). Most amino acids in the Manesh215 dataset were predicted with an MAE <1%, with tyrosine, histidine, tryptophan and phenylalanine predicted with <2% MAE.

## 3. Discussion

To improve the performance on different combinations of sequence-derived features, we validated five types of input variables using the TR7000 dataset for training and an independent test set (TS261). The performance of the method on different feature combinations was compared, with the results (Table 6) indicating that all five features contributed to RSA prediction.

When bidirectional information from a hidden node is merged in the BRNN , concatenation (concat) [51] and sum [47] are commonly used. In the SDBRNN, three merging operators (“concat”, “sum” and “weighting sum”) were used in different hidden layers. To assess the generalization of this BRNN model using hybrid merging operators, we compared the SDBRNN with a BLSTM using the “concat” operator (BLSTM_C), with an BLSTM using the “sum” operator (BLSTM_S) and with a unidirectional LSTM with 800 hidden nodes. Hyperparameters for the BLSTM_C and BLSTM_S were the same as those used for the SDBRNN, and rASA prediction was used to compare the different models. The results listed in Table 7 show that hybrid merging operators effectively promoted better predictive performance.

## 4. Materials and Methods

### 4.1. Datasets and Input Features

A large, non-homologous sequence dataset, produced using the PISCES server [52], was obtained. Structures exhibited <25% similarity and showed a resolution >3.0 Å, with R factors of 1.0. Three-dimensional structure files were downloaded from the RCSB Protein Data Bank. After removing redundancy in the test datasets using cd-hit [53], 7361 proteins (CullPDB7361) comprising 1,596,728 residues in proteins with sequence lengths between 50 and 600 residues were retained. Among these, 7000 proteins (TR7000) were used for training, and 261 proteins (TS261) and 100 proteins (VD100) were randomly selected for testing and validating the model. In addition to CullPDB7361, three public testing datasets, CB502, Manesh215 and CASP10, were used to evaluate the performance of our model. CB502 (82,420 residues) was selected from CB513 [54] and ordered by sequence length from long to short. The Manesh215 dataset [25] contains 47,243 residues. CASP10 from the Protein Sequence Prediction Center (http://predictioncenter.org/) has 123 domain fragments and extracts from 103 chains. The CASP10 dataset was selected according to protein identity and contained 20,778 residues from 90 proteins. The sequence lengths of the proteins in test datasets were all ≤600.

Five types of features, PSSM, protein sequence coding, conservation score, physical properties and physicochemical characteristics, were used as input features. PSSM-derived features have been widely used to perform protein-related predictions. PSSMs provide the effective frequency of occurrence of all 20 amino acid residues at each position of the sequence. PSSMs can be obtained by performing multiple sequence alignments on a large protein database (NCBI NR database) using PSI-BLAST [55]. PSI-BLAST involves an iterative search process in searching the profile of a query protein. The expectation value (E-value) and the number of iterations for PSI-BLAST are set to 0.001 and three, respectively. The PSSM profile is in the form of a 20×L matrix where *L* is the length and each amino acid in the sequence is described by 20 features.

Physical properties [40] include: a steric parameter (graph-shape index), polarizability, volume (normalized van der Waals volume), hydrophobicity, isoelectric point, helix probability and sheet probability. Specific values were derived from the study [40]. Protein physicochemical characteristics [56] include the number of atoms, electrostatic charges and potential hydrogen bonds.

To ensure smooth transitions in changes to the network gradient, the above features were normalized using a logistic regression function $y=1/(1+e^{-x})$.

Residue conservation was derived from the amino acid frequency distribution in the corresponding column of a multiple-sequence alignment of homologous proteins. A one-dimensional conservation score was computed according to Quan’s [41] previously described Equation (5):

(5) $$\begin{array}{c} R=\log20+\sum_{i=1}^{20}Q_{i}\log Q_{i} \end{array}$$

Commonly-used protein coding involves an orthogonal code. In addition to the 20 known residues, “X” represents unknown residues, and “NoSeq” represents non-protein sequences in our coding scheme. A 22×L matrix represents a sequence, where *L* is the sequence length and a 22-dimensional (dim) vector represents a residue in the sequence. However, the 22-dimensional coding vector represents a parsed, one-hot vector, where only one of 22 elements is a non-zero value. For instance, for the residue “A“, the coding is: “1 0 0 0 0 0 0 0 0 0 0 0 0 0 0 0 0 0 0 0 0 0“. We adopted an embedding operation from natural-language processing to transform sparse sequence features into denser representations. This embedding operation was implemented as a simple auto-encoder (a feed-forward neural-network layer) along with an embedding matrix mapping a sparse 22-dimensional vector into a denser 22-dimensional vector. This transformation was just converted into a one-hot vector, which coded one residue into a dense vector.

In our scheme, an input residue was represented by 53-dimensional features: 20-dim PSSM, 7-dimensional physical properties, 3-dimensional physicochemical characteristics, 1-dimensional conservation score and 22-dimensional protein codings. These features are all derived from the protein sequence.

The rASA of a residue in a protein chain is calculated by dividing the accessible surface area derived from DSSP [44] by the maximum solvent accessibility. However, there is no standard for maximum solvent accessibility. According to previous results [22,25], Gly-X-Gly extended tripeptides were used.

### 4.2. BRNN and Merging Operator

For sequence data $X=(x_{1},x_{2},x_{3}\ldots x_{t-1},x_{t},x_{t+1}\ldots x_{n})$, where $x_{i}$ is context dependent and strongly reliant on forward and backward information. The label vector $Y=(y_{1},y_{2},y_{3}\ldots y_{t-1},y_{t},y_{t+1}\ldots y_{n})$ is the target output space. Compared to a forward neural network, the current output from the recurrent neural network will be reverted backward for subsequent time-specific inputs. The recurrent neural network structure can be described as Equation (6):

(6) $$\begin{array}{c} h_{t}=f\left(W_{x}x+W_{h}h_{t-1}+b_{n}\right)\\ y_{t}=\sigma \left(W_{y}h_{t}+b_{y}\right) \end{array}$$

At the time T=t, the recurrent network can remember the information from previous $x_{1},x_{2},x_{3}\ldots x_{t-1}$ and the present input $x_{t}$. However, in many applications, the output $y_{t}$ might be dependent on the entire input sequence, as in protein sequences and handwriting-recognition examples. BRNN [57] combines an RNN that moves forward through time beginning from the start of the sequence along with another RNN that moves backward through time beginning from the end of the sequence. In this BRNN, time-increasing input is represented by $\vec{f}\left(x_{1},x_{2},x_{3},\ldots ,x_{t}\right)$, and time-decreasing input is represented by $\overset{\leftarrow }{f}\left(x_{t},x_{t+1},\ldots ,x_{n}\right)$. Bidirectional information is merged as the current node’s output. Therefore, BRNN is more suitable for context-related applications, and it is capable of outperforming unidirectional recurrent neural network. The BRNN can be described as Equation (7):

(7) $$\begin{array}{c} O_{t}=\eta \left(F_{t},B_{t},x\right)\\ s.t.\;F_{t}=\left(\vec{h_{1}},\vec{h_{2}},\ldots ,\vec{h_{t}}\right),\\ \;B_{t}=\left(\overset{\leftarrow }{h_{t}},{\overset{\leftarrow }{h}}_{t+1},\ldots ,\overset{\leftarrow }{h_{n}}\right) \end{array}$$

**Figure 4:**
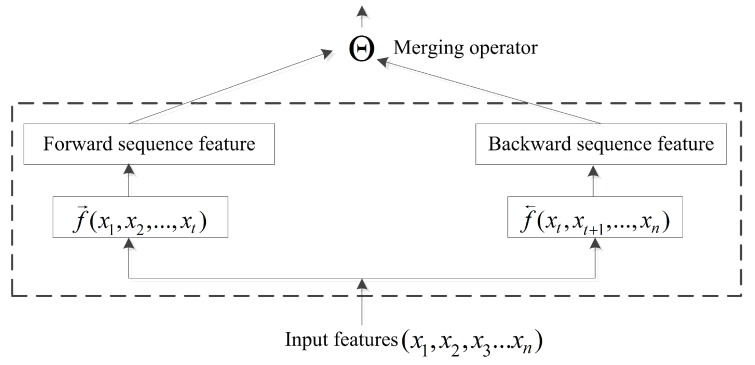
In order to remember more long-range information in the sequence study, when the past computing information and the future computing information are merged, the merging operator is proposed to execute the merging operation.

The BRNN structure is capable of efficiently learning sequence information [45]. Previous studies focused primarily on the BRNN methodology with little research on incorporating bidirectional-information merging. In a conventional neural network, pooling operations are computationally important, with the pooling layer responsible for accumulating convolutional results. The merging operator in a BRNN acts similarly to pooling operations. At time T=t, input forwarded to the current node is represented by $\vec{f}\left(x_{1},x_{2},x_{3},\ldots ,x_{t}\right)$, and the backward input is represented by $\overset{\leftarrow }{f}\left(x_{t},x_{t+1},\ldots ,x_{n}\right)$. Therefore, a common formula for the output of the current node is presented in Figure 4 and as follows:

(8) $$\begin{array}{c} h_{t}=W_{f}F_{t}\Theta W_{b}B_{t}+\gamma b_{t}\\ W_{f},W_{b}\in \left[0,1\right],\gamma \in \left\{0,1\right\} \end{array}$$

where Θ is the merging operator, Θ∈R,R={⨂,+,⨁,∞,max,min,avg,…}. ⨂ represents element-wise multiplication; + is the sum; ⨁ is the element-wise weighting sum; ∞ is concatenation; and ⨀ is the reshape operator. The merging operator can perform more computations than necessary when the information is merged via the aggregation operation.

### 4.3. Model

#### 4.3.1. SDBRNN

Protein primary structure represents a strongly time-ordered conformation, suggesting that it contains adequate information for the protein to fold into its native conformation. To capture more features in the primary structure, we use three types of computing operators in the merging operation. During the initial period, the computing node in the BRNN needs to remember backward, forward and current input information; therefore, the merging operator “concat” is used in the first layer. In the second layer, the computing node no longer needs to remember previous and future information; however, the “sum” operator reinforces bidirectional input aggregation, with bidirectional information again aggregated in the third layer. Therefore, a “weighting sum” operator is used for filtering unnecessary information and extracting key features. The equations associated with these operations are as follows (9)–(11):

(9) $$\begin{array}{c} h_{t}^{1}=F_{t}^{1}\infty B_{t}^{1} \end{array}$$

(10) $$\begin{array}{c} h_{t}^{2}=F_{t}^{2}+B_{t}^{2} \end{array}$$

(11) $$\begin{array}{c} h_{t}^{3}=W_{f}F_{t}^{3}+W_{b}B_{t}^{3}+b_{t}\\ O\left(h_{t}\right)=tanh\left(h_{t}^{3}\right) \end{array}$$

The recurrent neural network accepts a time-stepping sequence that makes the network extremely deep, with the depth making the network difficult to train because of the exploding or vanishing gradient [46]. Long short-term memory (LSTM) [58], which consists of a variety of gate structures (a forget gate, an input gate and an output gate) and a memory cell are used to address the vanishing gradient problem. In the SDBRNN architecture, the unidirectional computing node is performed by LSTM. The widely-accepted LSTM definition was provided by Graves [59], and the details of LSTM are described in Equation (12), which involves no peep-hole connections, mainly to improve computing performance.

(12) $$\begin{array}{c} i_{t}=\sigma \left(W_{i}x_{t}+U_{i}h_{t-1}+b_{i}\right)\\ f_{t}=\sigma \left(W_{f}x_{t}+U_{f}h_{t-1}+b_{f}\right)\\ o_{t}=\sigma \left(W_{o}x_{t}+U_{o}h_{t-1}+b_{o}\right)\\ c_{t}=f_{t}c_{t-1}+i_{t}\tanh\left(W_{c}x_{c}+U_{c}h_{t-1}+b_{c}\right)\\ h_{t}=o_{t}⨀\tanh\left(c_{t}\right) \end{array}$$

where σ is the sigmoid function; i,f,o and *c* are respectively the input gate, forget gate, output gate and cell activation vectors, all of which are the same size as the hidden vector *h*.

**Figure 5:**
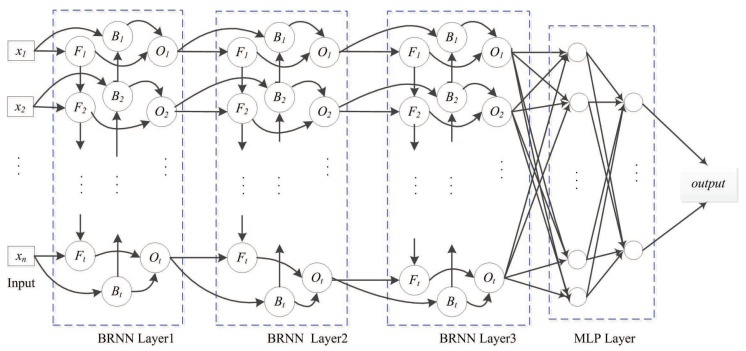
SDBRNN architecture. The merging operator “concat” is used in the first BRNN layer. The “sum” and “weighting sum” operator are used in the second and third layer. Two multi-perception networks are connected to BRNN.

The following BRNN layer represents a multi-layer perceptron (MLP) network that reduces the network scale. The MLP layer with logistic activation ultimately outputs the prediction, with logistic regression used for fitting the relative surface-area value. The SDBRNN architecture is shown in Figure 5. The cross-entropy loss function is used for model training:

(13) $$\begin{array}{c} L\left(x_{i}\right)=\frac{1}{N}\sum_{i=1}^{N}y_{i}\times log\left(\hat{y_{i}}\right) \end{array}$$

where $\hat{y_{i}}$ are the predicted probabilities of secondary structure labels, $y_{i}$ represent ground-truth labels of the secondary structure and *N* is the number of input residues. $\hat{y_{i}}$ is a vector of output activations prior to normalization with the logistic function. These derivatives are then fed back through the network using back propagation through time to determine the weight gradient.

In our model, rASA prediction is simulated as a regressive problem. With the predicted rASA values, the classifications are carried out in two states (buried and exposed). We have tried seven thresholds of 5, 10, 20, 25, 30, 40 and 50% in the two-state classification.

#### 4.3.2. Model Hyperparameters

SDBRNNs have three BRNN layers and two MLP layers. The first BRNN layer has 300 nodes, with 500 nodes in the other two layers. The first MLP layer has 128 nodes, and the second has 60 nodes. The Adam optimizing function was used for training the network using default settings, with the default learning rate set to 0.0008. This was reduced by 50%, whereas the validation accuracy decreased by more than 10-times. The threshold of the learning rate was set to 0.0001.

A natural stopping policy was accepted while the validation stopped decreasing. To balance model performance between fitting and generalization, a regularization or penalty term was attached to the loss function (e.g., L1 or L2). Unlike other models, regularization was not attached, and our experiment displayed dropout efficiency [60]. Except for the classification layer, each layer was regularized according to a dropout (*p* = 0.5) to avoid overfitting. Our model was implemented in Keras, which is a public deep learning software based on Theano [61]. Weights in the SDBRNN were initialized with default values in Keras. The network was trained using a single NVIDIA Tesla K40 GPU with 12 GB memory. Training the model requires about 16.5 min per epoch, and it takes about 0.04 seconds to test one sequence.

Proteins shorter than 600 AA were padded with all-zero features. The outputs corresponding to padded inputs are labeled as 0.5 according to the logistic function. The advantage of padding proteins is that it enables training the model on a GPU in batches.

## 5. Conclusions

Accessible RSA is often used as an important measure in proteomics study for describing protein properties. Compared with traditional machine learning techniques, deep learning exhibits wider generalization and is applied in our study to predict RSA area. Deep neural networks are stacked by various complicated neural networks, and more generalized representation capacity can be obtained. However, the deep neural networks own enormous parameters and need more samples and computing resources to train models. Use of the merging operator was proposed for merging computations in the BRNN hidden layer. We redesigned BLSTM merging using three types of merging operators (“concat”, “sum”, and “weighting sum”) in SDBRNN. Compared with the commonly-used BLSTM method using a single merging operator, as well as other predictors, SDBRNN captured more protein features and was more generalizable. Our results on test datasets verified this.

Relative solvent-accessible areas are greatly affected by maximum solvent-accessible areas, as more maximum solvent-accessible areas will lead to lower relative solvent-accessible areas and lower MAEs. However, there are no standard maximum solvent accessible areas. In this work, a novel deep learning method (SDBRNN) was presented for the prediction of RSA area, as well as for binary state classification. Our methods can be applied to a broad range of problems within and outside of computational biology.

## Figures and Tables

**Table 1:** The recent developments, in chronological order, for predicting the values of rASA.

Work	Algorithm	Description of Features	MAE (%)
Ahmad, 2003 [10]	Neural network	Amino acid composition	18.8
Wang, 2005 [18]	Multiple linear regression	PSSM	16.2
Garg, 2005 [13]	Neural network	PSSM, secondary structure	16.6
Nguyen, 2006 [27]	Two-stage SVR	PSSM	15.7
Wang, 2007 [17]	Support vector machine	PSSM	15.1
Dor, 2007 [14]	Neural network	PSSM, physical properties	14.3
Chang, 2008 [28]	Support vector regression	Enhances PSSM-based features	14.8
Faraggi, 2009 [15]	Neural networks	PSSM, physical properties, secondary structure	11.1
Meshkin, 2009 [29]	Pace regression	PSSM	13.4
Joo, 2012 [20]	k-nearest neighbor	PSSM	14.8
Kashefi, 2013 [30]	SVR and scatter search methods	PSSM, qualitative physicochemical features	12.31
Zhang, 2015 [24]	Weighted sliding window	PSSM, secondary structure, native disorder, physicochemical propensities, sequence-based features	14
Fan, 2016 [22]	Gradient boosted regression trees	PSSM, secondary structure, native disorder, conservation score, side-chain environment	9.4

The MAEs reported in this table were evaluated on a different dataset.

**Table 2:** Performance comparison in predicting relative solvent-accessible areas (the best results are shown in bold).

Method	CB502		Manesh215	
	MAE (%)	PCC	MAE (%)	PCC
SARpred	17.4	0.6	16.6	0.61
SVR	14.8	0.68	14.2	0.69
Real-SPINE	14.5	0.68	13.8	0.7
NetSurfP	14.3	0.71	13.6	0.7
PredRSA	9.4	0.73	9.0	0.75
SDBRNN	**8.8**	**0.75**	**8.2**	**0.78**

**Table 3:** Binary classification prediction comparison between our method and other reported methods with different thresholds on the Manesh215 dataset.

Method	Accuracy for Two-State Prediction						
	5%	10%	20%	25%	30%	40%	50%
SARpred	74.9	77.2	77.7	-	77.8	78.1	80.5
PR	76.8	74.8	75.3	76.7	77.7	79.8	86.3
SVR	80.9	80.1	78.7	-	-	-	80.8
SS-SVM	79.2	78.2	77.6	77.6	77.5	79.7	86.5
Two-stage SVR	81.1	78.7	77.6	77.3	-	-	79.5
PredRSA	80	81.6	80.9	81.1	82.2	87.1	**93.2**
SDBRNN	**83.5**	**82.4**	**82.3**	**82.6**	**83.5**	**87.4**	93

**Table 4:** Accuracy (%) performance comparison in binary classification prediction (the best results are shown in bold).

Threshold (%)	Manesh215		CB502		CASP10	
	PredRSA	SDBRNN	PredRSA	SDBRNN	PredRSA	SDBRNN
5	80.1	**83.5**	77.9	**82.3**	78.5	**84**
10	81.7	**82.4**	79	**80.9**	79.1	**82.1**
20	81	**82.3**	80.5	**80.7**	78.3	**80.6**
25	81.2	**82.6**	81	**81.4**	79.7	**80.2**
30	82.4	**83.5**	82.1	**82.5**	80.5	**81.2**
40	87.1	**87.4**	86.8	**87**	85	**85.4**
50	**93.2**	93	**93**	92.4	91.2	**91.4**

**Table 5:** Matthews’ correlation coefficient performance comparison in binary classification prediction.

Threshold (%)	Manesh215		CB502		CASP10	
	PredRSA	SDBRNN	PredRSA	SDBRNN	PredRSA	SDBRNN
5	0.54	**0.62**	0.5	**0.59**	0.48	**0.61**
10	0.63	**0.64**	0.58	**0.61**	0.57	**0.63**
20	0.61	**0.63**	0.6	0.6	0.56	**0.61**
25	0.58	**0.61**	0.57	**0.58**	0.56	**0.57**
30	0.54	**0.58**	0.52	**0.55**	0.51	**0.53**
40	0.42	**0.48**	0.39	**0.46**	0.4	**0.43**
50	0.25	**0.34**	0.23	**0.33**	0.3	**0.31**

**Table 6:** Different combinations of sequence-derived features for SDBRNN predictors on an independent test set (TS261).

Feature	MAE (%)	PCC
PSSM	9.33	0.732
PSSM + SC	9.03	0.749
PSSM + SC + CS	9.00	0.750
PSSM + SC + CS + PP	8.95	0.750
PSSM + SC + CS + PP + PC	**8.86**	**0.753**

PSSM: position specific scoring matrix, 20 dimensions; SC: protein sequence coding, 22 dimensions; CS: residue conservation score, 1 dimension; PP: physical properties, 7 dimensions; PC: physicochemical characteristics, 3 dimensions.

**Table 7:** Comparison of different LSTM models on relative RSA area prediction. MAE is the value percentage (%).

Method	CB502		Manesh215		CASP10		TS261	
	MAE (%)	PCC	MAE (%)	PCC	MAE (%)	PCC	MAE (%)	PCC
LSTM	9.8	0.694	9.4	0.722	10.0	0.698	10.0	0.695
BLSTM_C	9.0	0.74	8.44	0.772	9.33	0.734	9.0	0.748
BLSTM_S	8.93	0.744	8.33	0.775	9.26	0.739	8.96	0.747
SDBRNN	**8.84**	**0.748**	**8.24**	**0.777**	**9.19**	**0.742**	**8.86**	**0.753**

## Supplementary Materials

The following are available online at http://www.mdpi.com/2218-273X/8/2/33/s1, File f1: PDB-ID list of CullPDB7361. Table t1: Predicted MAE and accuracy at the threshold of 25% of individual sequences on the Manesh215 dataset.

## Abbreviations

The following abbreviations are used in this manuscript:

RSA	Residue Solvent Accessibility
rASA	relative accessible solvent area
BLSTM	bidirectional long-short memory
SDBRNN	stacked deep bidirectional recurrent neural network
RNN	recurrent neural network
BRNN	bidirectional recurrent neural network
LSTM	long short-term memory
MLP	multi-layer perceptron
MAE	mean absolute error
PCC	Pearson’s correlation coefficient
MCC	Matthews’ correlation coefficient
ACC	Accuracy
