# Supplementary material for: Protein Solvent-Accessibility Prediction by a Stacked Deep Bidirectional Recurrent Neural Network
Source: Biomolecules. 2018 May 25;8(2):33. doi: 10.3390/biom8020033 (PMC6023031; doi:10.3390/biom8020033)
Supplement: Supplementary file 1 [file biomolecules-08-00033-s001.pdf]

File f1: PDB-ID list of CullPDB7361

16VPA	1A0AA	1A0IA	1A41A	1A5TA	1A62A	1A73A	1A92A	1AE9A
1AEPA	1AH7A	1AHSA	1AIHA	1ALUA	1AM7A	1AOLA	1ATGA	1ATZA
1AU7A	1AVOA	1AYOA	1AZOA	1B33N	1B35A	1B35B	1B35D	1B3TA
1B5LA	1B6AA	1B77A	1B89A	1B8KA	1B9LA	1B9WA	1BCOA	1BCPB
1BCPD	1BCPF	1BEAA	1BG1A	1BGFA	1BH9B	1BJAA	1BKRA	1BL0A
1BM8A	1BOOA	1BOWA	1BRTA	1BTKA	1BVOA	1BW0A	1BX7A	1BXYA
1BYIA	1C1KA	1C1LA	1C1YB	1C30B	1C3CA	1C4OA	1C5EA	1C6VX
1C8BA	1C8NA	1C9BA	1C9KA	1CC8A	1CCWA	1CCWB	1CF7B	1CFRA
1CFZA	1CI4A	1CIDA	1CJAA	1CL8A	1CMCA	1CNT1	1COLA	1COZA
1CQ3A	1CR1A	1CV8A	1CVJA	1CXQA	1CXZB	1CY5A	1CZNA	1D02A
1D0DA	1D0QA	1D2OA	1D2SA	1D3BB	1D3YA	1D4OA	1D4TA	1D7MA
1D8HA	1D8WA	1DC1A	1DCFA	1DCSA	1DD9A	1DDLA	1DDWA	1DEBA
1DFMA	1DFUP	1DG6A	1DGWA	1DGWX	1DGWY	1DJ0A	1DJ7A	1DJ7B
1DJ8A	1DK8A	1DKGA	1DMGA	1DMLA	1DMUA	1DOWA	1DP4A	1DP7P
1DQPA	1DS1A	1DTDB	1DUSA	1DVKA	1DVOA	1DWKA	1DYOA	1DZFA
1E29A	1E2WA	1E3JA	1E44B	1E58A	1E5KA	1E6UA	1E7LA	1E9RA
1EAQA	1EARA	1EAYC	1EB6A	1EBFA	1EBPA	1EDZA	1EERA	1EEXA
1EEXB	1EEXG	1EF1C	1EF8A	1EFDN	1EG2A	1EGWA	1EI7A	1EI9A
1EJDA	1EJFA	1EK9A	1EKQA	1EL6A	1ELKA	1ELUA	1ELWA	1EM2A
1EOKA	1EP3B	1EPUA	1EQ2A	1ES5A	1ES9A	1ETEA	1EUVA	1EUWA
1EV7A	1EVLA	1EVSA	1EW4A	1EWFA	1EXTA	1EZ0A	1EZ3A	1EZGA
1EZJA	1EZWA	1F00I	1F0XA	1F1EA	1F1MA	1F20A	1F32A	1F35A
1F3UA	1F3UB	1F3VA	1F46A	1F5NA	1F5QB	1F5VA	1F60B	1F86A
1F89A	1F94A	1F9VA	1FC3A	1FCDA	1FCQA	1FCYA	1FEUA	1FIOA
1FIPA	1FIUA	1FJRA	1FK5A	1FKMA	1FLOA	1FM0D	1FM0E	1FN9A
1FO8A	1FOBA	1FP2A	1FPZA	1FS7A	1FT5A	1FTRA	1FX2A	1FXKA
1FXKC	1FYEA	1FYHA	1G12A	1G2RA	1G31A	1G3KA	1G3PA	1G55A
1G5HA	1G5TA	1G61A	1G66A	1G73A	1G8EA	1G8QA	1GA6A	1GA8A
1GAKA	1GD2E	1GD8A	1GK9A	1GK9B	1GKMA	1GL2C	1GMLA	1GMUA
1GMXA	1GNLA	1GNWA	1GNYA	1GO3F	1GO4E	1GOTG	1GP0A	1GPRA
1GQ8A	1GQEA	1GS5A	1GS9A	1GU2A	1GU3A	1GU4A	1GU7A	1GUQA
1GUTA	1GV9A	1GVNA	1GWEA	1GWMA	1GWUA	1GXCA	1GXMA	1GXRA
1GXSB	1GXUA	1GY7A	1GYXA	1GZSB	1H03P	1H0HB	1H1NA	1H21A
1H2CA	1H2IA	1H2SB	1H3GA	1H4RA	1H4UA	1H4XA	1H5WA	1H6FA
1H72C	1H8PA	1H97A	1H99A	1H9AA	1HBNA	1HBNB	1HBNC	1HCNB
1HDHA	1HDOA	1HDRA	1HE1A	1HEKA	1HF2A	1HFES	1HH8A	1HI9A
1HKQA	1HLMA	1HO8A	1HP1A	1HQ0A	1HQZ1	1HRUA	1HSTA	1HULA
1HUWA	1HUXA	1HX6A	1HX8A	1HXIA	1HXRA	1HYOA	1HZ4A	1HZTA
1I0RA	1I1JA	1I1RB	1I1WA	1I24A	1I27A	1I2KA	1I2TA	1I36A
1I3CA	1I3JA	1I4DA	1I4JA	1I4UA	1I58A	1I60A	1I71A	1I7QA
1I88A	1I8AA	1I8NA	1IAPA	1IARB	1ID0A	1ID1A	1IE7C	1IFQA
1IFRA	1IG0A	1IGNA	1IGQA	1IGWA	1II5A	1IIBA	1IJBA	1IJYA

1IK9A	1IM3D	1IN0A	1IN4A	1INLA	1IO0A	1IOMA	1ITHA	1ITVA
1ITXA	1IUQA	1IX9A	1IXMA	1IZMA	1IZOA	1J0PA	1J1TA	1J24A
1J27A	1J3AA	1J3WA	1J5YA	1J6RA	1J77A	1J7XA	1J83A	1J8BA
1J8RA	1J98A	1JADA	1JB0D	1JB0E	1JB0F	1JB0L	1JB7B	1JCDA
1JDWA	1JE5A	1JETA	1JEYA	1JEYB	1JF3A	1JFBA	1JFIA	1JG1A
1JG5A	1JH6A	1JHJA	1JHSA	1JI7A	1JIDA	1JIWI	1JIXA	1JK0B
1JK3A	1JKEA	1JL0A	1JLYA	1JMVA	1JNDA	1JNIA	1JO0A	1JOSA
1JOVA	1JQ5A	1JQLB	1JR2A	1JR7A	1JS8A	1JSSA	1JSUC	1JUHA
1JX6A	1JX7A	1JY1A	1JY2O	1JYAA	1JYEA	1JYHA	1JYOA	1JYOE
1JZTA	1K04A	1K1FA	1K3SA	1K3XA	1K4IA	1K4ZA	1K5CA	1K5NA
1K6XA	1K77A	1K78A	1K7CA	1K7JA	1K7WA	1K8KC	1K8KD	1K8KE
1K8KF	1K8KG	1K8RB	1K8TA	1K8WA	1KA1A	1KA8A	1KAEA	1KAFA
1KCFA	1KCGC	1KCMA	1KCQA	1KCZA	1KF6C	1KF6D	1KGDA	1KHXA
1KHYA	1KI9A	1KJNA	1KJQA	1KLOA	1KMIZ	1KMTA	1KNGA	1KNXA
1KNZA	1KOEA	1KP6A	1KQ6A	1KQFC	1KQPA	1KQRA	1KS8A	1KS9A
1KT6A	1KTGA	1KU3A	1KVEA	1KVEB	1KWAA	1KXOA	1KXPD	1KYFA
1KYQA	1KZFA	1KZQA	1L0SA	1L1OC	1L2WI	1L3KA	1L3LA	1L3PA
1L6PA	1L6RA	1L7AA	1L8DA	1L8WA	1L9LA	1LBVA	1LC0A	1LC5A
1LDDA	1LEHA	1LFBA	1LFDA	1LFPA	1LGHA	1LJ2A	1LKTA	1LM5A
1LMIA	1LMLA	1LO7A	1LQTA	1LQVA	1LS1A	1LSHB	1LUCA	1LUZA
1LWBA	1LXJA	1LYQA	1LZLA	1M0DA	1M0KA	1M0UA	1M0WA	1M15A
1M1EB	1M1FA	1M1HA	1M1QA	1M22A	1M2DA	1M3YA	1M40A	1M4IA
1M4JA	1M4UA	1M55A	1M7JA	1M93B	1M9ZA	1MDAH	1MDLA	1MG4A
1MG7A	1MIJA	1MIWA	1MIXA	1MJ4A	1MJ5A	1MJNA	1MKFA	1MKKA
1MN8A	1MNAA	1MNNA	1MR1C	1MSCA	1MSPA	1MTPA	1MTYB	1MTYD
1MUNA	1MUSA	1MUWA	1MW5A	1MW9X	1MWPA	1MWQA	1MY7A	1N08A
1N13B	1N1CA	1N1FA	1N1JA	1N2ZA	1N3LA	1N40A	1N4WA	1N5UA
1N62A	1N62C	1N7SC	1N7VA	1N7ZA	1N81A	1N8VA	1N93X	1NBWB
1NC5A	1NEPA	1NG0A	1NG6A	1NGMB	1NH1A	1NH2C	1NH2D	1NHYA
1NIGA	1NIJA	1NKDA	1NKGA	1NKIA	1NKZA	1NLQA	1NLWA	1NMOA
1NNFA	1NNWA	1NNXA	1NO1A	1NO7A	1NP6A	1NQJA	1NQKA	1NRJA
1NRJB	1NSZA	1NTHA	1NTYA	1NU0A	1NUYA	1NWWA	1NXHA	1NXMA
1NYCA	1NZ0A	1NZJA	1NZYB	1O13A	1O22A	1O4WA	1O59A	1O65A
1O66A	1O6AA	1O6DA	1O70A	1O75A	1O7DE	1O7IA	1O7JA	1O82A
1O88A	1O9YA	1OA8A	1OAIA	1OAPA	1OBBA	1OCYA	1OD3A	1OD6A
1ODHA	1ODMA	1OE4A	1OEYA	1OEYJ	1OF5B	1OF8A	1OFCX	1OFLA
1OFUX	1OGDA	1OGOX	1OH0A	1OHUA	1OI0A	1OI2A	1OI7A	1OIHA
1OISA	1OJ5A	1OJHA	1OKCA	1OKGA	1OKSA	1OLTA	1OMZA	1OO0A
1OQJA	1OR4A	1ORSC	1ORUA	1OSYA	1OTKA	1OU8A	1OW1A	1OW4A
1OZ9A	1OZJA	1P1JA	1P1MA	1P1XA	1P32A	1P35A	1P3CA	1P3DA
1P3RA	1P4CA	1P4XA	1P5DX	1P5VB	1P5ZB	1P6OA	1P90A	1P9LA
1P9YA	1PAQA	1PBJA	1PBWA	1PBYA	1PBYC	1PC3A	1PC6A	1PCFA
1PD3A	1PFBA	1PG6A	1PJHA	1PKHA	1PL3A	1PM4A	1PMHX	1PO5A
1POIB	1PP0A	1PPJB	1PPJD	1PSRA	1PSWA	1PU6A	1PUCA	1PV5A

1PX5A	1PXUA	1PXZA	1PYAA	1PYAB	1PZ4A	1PZWA	1Q06A	1Q0PA
1Q0RA	1Q15A	1Q1FA	1Q1HA	1Q2HA	1Q35A	1Q5YA	1Q5ZA	1Q67A
1Q6ZA	1Q7EA	1Q7FA	1Q7LA	1Q7LB	1Q87A	1Q8CA	1Q8DA	1Q9JA
1Q9UA	1QAZA	1QCSA	1QD1A	1QD6C	1QEXA	1QF8A	1QFJA	1QFTA
1QG8A	1QGEE	1QHDA	1QHIA	1QHXA	1QJPA	1QKRA	1QLWA	1QNRA
1QO7A	1QQ5A	1QQEA	1QQFA	1QQP1	1QQRA	1QR0A	1QSMA	1QTFA
1QUUA	1QV9A	1QW2A	1QW9A	1QWGA	1QWOA	1QWRA	1QYIA	1QYNA
1QYSA	1QZMA	1R0DA	1R29A	1R44A	1R4XA	1R5MA	1R6DA	1R6JA
1R6XA	1R71A	1R75A	1R7AA	1R7JA	1R8IA	1R9LA	1R9WA	1RA0A
1RCQA	1RCWA	1RDO1	1REGX	1REPC	1RFYA	1RGXA	1RH6A	1RIFA
1RKIA	1RL6A	1RLWA	1RLZA	1RO2A	1RO7A	1ROCA	1RP3B	1RR7A
1RSSA	1RT8A	1RTQA	1RTTA	1RTWA	1RU4A	1RV9A	1RW1A	1RWJA
1RWRA	1RWZA	1RX0A	1RXQA	1RY9A	1RYLA	1RYOA	1RYPK	1RYPL
1RZHH	1RZHM	1S0PA	1S12A	1S1DA	1S21A	1S29A	1S2XA	1S3CA
1S48A	1S4CA	1S4KA	1S5DA	1S7IA	1S7ZA	1S98A	1S99A	1S9RA
1SA3A	1SAUA	1SAZA	1SBXA	1SBYA	1SCFA	1SD4A	1SDDA	1SDIA
1SEDA	1SEFA	1SEIA	1SF8A	1SFPA	1SFXA	1SG4A	1SG6A	1SH8A
1SHUX	1SJWA	1SKVA	1SKZA	1SL8A	1SMXA	1SO4A	1SQ5A	1SQ9A
1SQSA	1SQWA	1SR4A	1SR8A	1SRQA	1STMA	1SUMB	1SURA	1SVFA
1SVMA	1SVSA	1SWVA	1SZ7A	1SZHA	1SZIA	1SZOA	1SZQA	1SZWA
1T0BA	1T0FA	1T0IA	1T0TV	1T1UA	1T1VA	1T3GA	1T3JA	1T3YA
1T4AA	1T4OA	1T4WA	1T61A	1T6AA	1T6DA	1T6LA	1T6SA	1T6UA
1T77A	1T8KA	1T8SA	1T92A	1T9IA	1TAFA	1TC3C	1TC5A	1TD6A
1TE2A	1TH7A	1THTA	1TIFA	1TJLA	1TKEA	1TL2A	1TP6A	1TQ5A
1TQGA	1TQYB	1TR0A	1TS9A	1TT8A	1TTZA	1TU9A	1TUAA	1TUKA
1TUOA	1TUWA	1TV8A	1TVXA	1TWDA	1TWFF	1TWFH	1TWFJ	1TWUA
1TXKA	1TXLA	1TZPA	1U02A	1U07A	1U0JA	1U0SA	1U14A	1U19A
1U2CA	1U2HA	1U2MA	1U3EM	1U5DA	1U5KA	1U5PA	1U5UA	1U5XA
1U60A	1U7GA	1U7IA	1U7KA	1U7LA	1U7ZA	1U84A	1U8SA	1U8VA
1U9LA	1UA4A	1UA7A	1UCDA	1UCRA	1UCSA	1UD9A	1UEBA	1UEKA
1UF5A	1UFYA	1UGIA	1UHVA	1UI0A	1UJ2A	1UJ8A	1UJWB	1UKFA
1UKKA	1UNNC	1UNQA	1UOYA	1UPKA	1UPTB	1UQTA	1URQA	1US0A
1USCA	1USGA	1USMA	1USUB	1USYC	1UTEA	1UTYA	1UUJA	1UUNA
1UUYA	1UV7A	1UW4A	1UW4B	1UWCA	1UWKA	1UX5A	1UX6A	1UYJA
1UYNX	1UZ3A	1UZXA	1V0AA	1V0WA	1V2BA	1V2XA	1V2ZA	1V30A
1V33A	1V4AA	1V4PA	1V5IB	1V5VA	1V6PA	1V6TA	1V72A	1V74A
1V74B	1V77A	1V7MV	1V84A	1V8HA	1V96A	1V9FA	1V9MA	1VA6A
1VAJA	1VBKA	1VBVA	1VBWA	1VCHA	1VD6A	1VDDA	1VDKA	1VE1A
1VE2A	1VE4A	1VG0A	1VGJA	1VH4A	1VH5A	1VHNA	1VI0A	1VI1A
1VI6A	1VJFA	1VJGA	1VJLA	1VJNA	1VJQA	1VJUA	1VK1A	1VK3A
1VKEA	1VKFA	1VKKA	1VKYA	1VL1A	1VL7A	1VLYA	1VMBA	1VMGA
1VMHA	1VP7A	1VP8A	1VPBA	1VPQA	1VPRA	1VQO1	1VQO3	1VQOA
1VQOB	1VQOH	1VQOL	1VQOM	1VQON	1VQOP	1VQOQ	1VQOS	1VQOT
1VQOU	1VQOV	1VQOW	1VQOX	1VQOZ	1VR4A	1VR7A	1VRBA	1VSGA

1VSRA	1VYBA	1VYIA	1VYKA	1VYRA	1VZYA	1W0HA	1W0NA	1W1HA
1W1WE	1W23A	1W2WA	1W2WB	1W33A	1W4SA	1W53A	1W5QA	1W5RA
1W5TA	1W6SA	1W6SB	1W8SA	1W99A	1W9YA	1W9ZA	1WB4A	1WBHA
1WC2A	1WCWA	1WD3A	1WDDA	1WDDS	1WDJA	1WERA	1WHZA	1WIWA
1WLFA	1WLGA	1WLJA	1WLZA	1WMHA	1WMHB	1WMIA	1WN2A	1WNAA
1WNYA	1WOCA	1WOLA	1WOUA	1WPBA	1WPNA	1WPUA	1WPXB	1WQ6A
1WRDA	1WS8A	1WT6A	1WTEA	1WTHD	1WTJA	1WUBA	1WURA	1WV3A
1WVFA	1WVGA	1WVHA	1WVQA	1WWBX	1WWIA	1WWJA	1WWMA	1WWPA
1WY2A	1WY6A	1WY9A	1WZ3A	1WZDA	1X0TA	1X1NA	1X3LA	1X54A
1X6IA	1X6MA	1X6OA	1X6ZA	1X7DA	1X8BA	1X8QA	1X8YA	1X91A
1X9IA	1X9ZA	1XAKA	1XAUA	1XAWA	1XCLA	1XCRA	1XD3A	1XDNA
1XDYA	1XE7A	1XFKA	1XG0A	1XG0C	1XG8A	1XHNA	1XIPA	1XIWA
1XJUA	1XJVA	1XK5A	1XKPA	1XKPB	1XKPC	1XKRA	1XKSA	1XKZA
1XL3C	1XLQA	1XLYA	1XMKA	1XMTA	1XMXA	1XNXA	1XO0A	1XO1A
1XODA	1XOUA	1XOUB	1XOVA	1XPPA	1XQAA	1XQOA	1XQRA	1XR4A
1XRSA	1XSVA	1XSZA	1XTPA	1XTTA	1XUBA	1XV5A	1XVSA	1XW3A
1XWVA	1Y0KA	1Y0NA	1Y12A	1Y14A	1Y1LA	1Y28A	1Y43B	1Y4MA
1Y5HA	1Y60A	1Y66A	1Y6XA	1Y6ZA	1Y71A	1Y8XB	1Y96A	1Y96B
1Y9BA	1Y9IA	1Y9LA	1YA5T	1YACA	1YARA	1YARO	1YAVA	1YB3A
1YBKA	1YCYA	1YD0A	1YD7A	1YDXA	1YE8A	1YF3A	1YFQA	1YGAA
1YGTA	1YHNB	1YHTA	1YI9A	1YISA	1YJ7A	1YK3A	1YKDA	1YKHA
1YKHB	1YKIA	1YLIA	1YLLA	1YLMA	1YLXA	1YM3A	1YN3A	1YNFA
1YNRA	1YOVA	1YOZA	1YP0A	1YPXA	1YPYA	1YQ5A	1YQGA	1YQSA
1YQZA	1YREA	1YT3A	1YT8A	1YTLA	1YU0A	1YUEA	1YUMA	1YVWA
1YW4A	1YX1A	1YY7A	1YZVA	1Z0NA	1Z0PA	1Z0WA	1Z1YA	1Z21A
1Z2NX	1Z2WA	1Z3EA	1Z3EB	1Z4RA	1Z67A	1Z6RA	1Z70X	1Z72A
1Z7MA	1Z8UA	1Z94A	1Z9LA	1ZA0A	1ZA7A	1ZB1A	1ZBOA	1ZBSA
1ZBXB	1ZC3B	1ZCEA	1ZEEA	1ZFNA	1ZGHA	1ZGKA	1ZHSA	1ZHVA
1ZI8A	1ZJCA	1ZK4A	1ZK5A	1ZK8A	1ZL0A	1ZLDA	1ZMTA	1ZN6A
1ZOXA	1ZPSA	1ZS3A	1ZS4A	1ZS9A	1ZSQA	1ZT3A	1ZTDA	1ZTHA
1ZUUA	1ZV1A	1ZVAA	1ZVPA	1ZVTA	1ZWWA	1ZXKA	1ZXXA	1ZY7A
1ZY9A	1ZYLA	1ZYMA	1ZYNA	1ZYOA	1ZYQA	1ZZ1A	1ZZKA	2A14A
2A15A	2A1FA	2A1HA	2A1IA	2A1JA	2A1KA	2A1RA	2A1VA	2A1XA
2A2FX	2A2KA	2A2MA	2A35A	2A3NA	2A4XA	2A5HA	2A5YB	2A5ZA
2A65A	2A67A	2A6HE	2A6SA	2A6ZA	2A72A	2A98A	2A9IA	2A9SA
2AAMA	2AB5A	2ABSA	2AEBA	2AEGA	2AG4A	2AGKA	2AH5A	2AHDA
2AHMA	2AJ7A	2AKZA	2AL6A	2AMHA	2AMYA	2ANEA	2ANUA	2ANXA
2AO9A	2AOTA	2AP3A	2APJA	2APLA	2AQ4A	2AQ6A	2AQWA	2ASBA
2ASKA	2ATZA	2AU5A	2AUWA	2AVDA	2AVUE	2AVWA	2AWIA	2AXOA
2AXWA	2AYDA	2AZ0A	2AZ4A	2AZEA	2AZEB	2AZNA	2B0AA	2B0VA
2B1YA	2B2AA	2B4AA	2B4HA	2B4JC	2B4VA	2B4WA	2B5IC	2B81A
2B82A	2B8IA	2B8MA	2B97A	2B99A	2B9DA	2B9SB	2BA2A	2BASA
2BB6A	2BBAA	2BBDA	2BBRA	2BCMA	2BDRA	2BE1A	2BE3A	2BF6A
2BFCA	2BFDB	2BGCA	2BGHA	2BH1A	2BH1X	2BHVA	2BHWA	2BIVA

2BJ0A	2BJFA	2BJIA	2BJNA	2BJQA	2BKFA	2BKRA	2BKXA	2BKYA
2BL0A	2BL2A	2BL8A	2BLLA	2BLNA	2BM5A	2BM8A	2BMOA	2BMOB
2BNLA	2BNMA	2BONA	2BOUA	2BPA1	2BPA2	2BPSA	2BRFA	2BS2C
2BSJA	2BSQE	2BT9A	2BU3A	2BWFA	2BWRA	2BYKA	2BZ1A	2BZ4A
2BZVA	2C0GA	2C0NA	2C1DA	2C1DB	2C1VA	2C1WA	2C24A	2C2IA
2C2QA	2C2UA	2C3VA	2C4JA	2C5KT	2C5LC	2C5RA	2C5UA	2C5WB
2C61A	2C71A	2C78A	2C7NA	2C8EE	2C8MA	2C92A	2C9WA	2CA6A
2CB8A	2CBZA	2CC6A	2CCMA	2CCQA	2CCVA	2CDCA	2CDUA	2CFQA
2CG7A	2CH7A	2CI1A	2CIBA	2CIUA	2CIWA	2CJ4A	2CKKA	2CLYA
2CLYB	2CLYC	2CMGA	2CMPA	2CMZA	2CNQA	2CO5A	2COVD	2CS7A
2CU3A	2CVEA	2CVIA	2CW6A	2CW9A	2CWRA	2CWSA	2CWYA	2CX1A
2CX6A	2CX7A	2CXAA	2CXHA	2CXIA	2CXNA	2CXYA	2CY5A	2CYJA
2CZLA	2CZSA	2CZVC	2D00A	2D0BA	2D0TA	2D1SA	2D28C	2D2SA
2D3DA	2D42A	2D48A	2D4PA	2D4XA	2D54A	2D59A	2D5FA	2D5MA
2D68A	2D74B	2D7EA	2D7IA	2D7VA	2D80A	2D8DA	2D9RA	2DB7A
2DBSA	2DCLA	2DDRA	2DDXA	2DDZA	2DE3A	2DE6A	2DEJA	2DF7A
2DG1A	2DGDA	2DH2A	2DI4A	2DJFA	2DKJA	2DKOA	2DKOB	2DLAA
2DLBA	2DOKA	2DOQD	2DPFA	2DPLA	2DPMA	2DQLA	2DQWA	2DRUA
2DS2B	2DSKA	2DSLA	2DSXA	2DSYA	2DT8A	2DTJA	2DU3A	2DVMA
2DWKA	2DWUA	2DXAA	2DXUA	2DY0A	2DYIA	2DYJA	2DYNA	2DYOA
2DYTA	2E11A	2E12A	2E1FA	2E1MB	2E1MC	2E2AA	2E2EA	2E2OA
2E3HA	2E3NA	2E4MC	2E4TA	2E52A	2E56A	2E5FA	2E5YA	2E67A
2E6MA	2E6XA	2E7VA	2E8BA	2E8EA	2E9XA	2E9XB	2E9XC	2E9XD
2EB4A	2EBEA	2ECEA	2ED6A	2EFEA	2EFJA	2EFKA	2EFVA	2EGVA
2EH3A	2EHPA	2EHWA	2EHZA	2EI9A	2EJXA	2EK0A	2EKDA	2ELCA
2ENGA	2EQBB	2ERFA	2ERVA	2ERWA	2ES4D	2ES9A	2ESSA	2ETJA
2EV1A	2EW0A	2EWTA	2EX2A	2EX3B	2EX5A	2EY4C	2EZ2A	2EZVA
2F01A	2F1FA	2F20A	2F22A	2F23A	2F2CA	2F31A	2F46A	2F48A
2F4IA	2F4MA	2F4NA	2F5GA	2F5JA	2F5TX	2F5UA	2F60K	2F62A
2F6MA	2F6MB	2F7BA	2F8LA	2F9FA	2FA1A	2FA8A	2FAOA	2FB0A
2FB5A	2FBAA	2FBIA	2FCAA	2FCJA	2FCKA	2FCLA	2FCOA	2FCTA
2FCWA	2FCWB	2FDNA	2FDOA	2FEFA	2FELA	2FEPA	2FEXA	2FFGA
2FFMA	2FFSA	2FGGA	2FGQX	2FGTA	2FHDA	2FHPA	2FHZA	2FHZB
2FI0A	2FI1A	2FI9A	2FIPA	2FIQA	2FIUA	2FIYA	2FJ8A	2FJI12FJRA
2FK6A	2FKCA	2FKKA	2FLIA	2FM9A	2FMAA	2FMYA	2FNAA	2FNJB
2FOZA	2FP1A	2FP4B	2FP8A	2FPHX	2FPNA	2FQ3A	2FR5A	2FREA
2FSQA	2FSUA	2FT0A	2FTWA	2FTXA	2FTXB	2FU2A	2FU4A	2FULA
2FUPA	2FURA	2FWHA	2FY7A	2FYGA	2FYUK	2FZFA	2FZSA	2FZTA
2G0CA	2G0DA	2G2CA	2G2QA	2G30A	2G3RA	2G3VA	2G3WA	2G40A
2G45A	2G5GX	2G6TA	2G7LA	2G7OA	2G7SA	2G8LA	2G8SA	2G9WA
2GA1A	2GAGC	2GAGD	2GAKA	2GAUA	2GB4A	2GBLA	2GBOA	2GD5A
2GDQA	2GEXA	2GF4A	2GF6A	2GGCA	2GHSA	2GHTA	2GHVC	2GIAA
2GIBA	2GIGA	2GIYA	2GJ2A	2GJLA	2GJXA	2GK9A	2GKEA	2GKGA
2GKPA	2GLFA	2GLZA	2GMQA	2GNOA	2GNXA	2GOMA	2GPIA	2GPJA

2GR8A	2GRCA	2GRRB	2GRVA	2GS5A	2GSCA	2GSOA	2GSVA	2GT1A
2GTIA	2GU3A	2GU9A	2GUDA	2GUFA	2GUIA	2GUJA	2GUKA	2GUZB
2GV5C	2GVIA	2GWDA	2GWFA	2GWMA	2GX5A	2GXQA	2GYQA	2GZ4A
2GZ6A	2GZSA	2H0QA	2H1CA	2H1TA	2H1VA	2H21A	2H4OA	2H5NA
2H62C	2H7ZA	2H88B	2H88C	2H88D	2H8EA	2H8GA	2H9AA	2HA8A
2HA9A	2HAZA	2HBAA	2HBOA	2HCFA	2HD9A	2HDIB	2HEOA	2HEUA
2HEWF	2HFEC	2HFNA	2HHCA	2HHZA	2HINA	2HIQA	2HIVA	2HJ1A
2HJEA	2HJMA	2HKVA	2HL0A	2HLJA	2HLRA	2HLYA	2HNGA	2HNUA
2HO0A	2HOXA	2HP0A	2HQ7A	2HQBA	2HQLA	2HQSA	2HQTA	2HQYA
2HRAA	2HS1A	2HSBA	2HSNA	2HTHB	2HU9A	2HUEB	2HUEC	2HUHA
2HW2A	2HWJA	2HX0A	2HX5A	2HXRA	2HY5A	2HY5C	2HY7A	2HYPA
2HYTA	2HZCA	2HZLA	2HZMA	2HZMB	2HZTA	2I00A	2I06A	2I0KA
2I0MA	2I15A	2I1SA	2I2CA	2I2LA	2I2XA	2I44A	2I46A	2I49A
2I53A	2I5HA	2I5IA	2I6HA	2I6XA	2I74A	2I7GA	2I7HA	2I7RA
2I7XA	2I8DA	2I8TA	2I9CA	2I9IA	2I9WA	2IA1A	2IA7A	2IAFA
2IAYA	2IAZA	2IB0A	2IBNA	2IC6A	2ICHA	2ICUA	2ICWG	2ID4A
2IDOB	2IF6A	2IG3A	2IGIA	2IGPA	2IGSA	2II0A	2II2A	2IIHA
2IJLA	2IJRA	2IKKA	2IKSA	2IL5A	2ILKA	2ILRA	2IM8A	2IMFA
2IMHA	2IMJA	2IMQX	2IMRA	2IMSA	2IN3A	2IN5A	2INUA	2INWA
2IOJA	2IP1A	2IP6A	2IQCA	2IQIA	2IT2A	2IT9A	2ITBA	2IUWA
2IUYA	2IVFC	2IW1A	2IWRA	2IX7C	2IXMA	2IXSA	2IY2A	2IYKA
2IYVA	2IZ6A	2IZRA	2IZWA	2J0AA	2J16A	2J1DG	2J1VA	2J3TC
2J43A	2J4DA	2J4OA	2J58A	2J6AA	2J6BA	2J6LA	2J73A	2J7QA
2J8AA	2J8BA	2J97A	2J9OA	2J9WA	2JA4A	2JA9A	2JBYA	2JC9A
2JD3A	2JDCA	2JDID	2JDIG	2JDIH	2JDJA	2JE3A	2JEEA	2JEKA
2JFRA	2JG0A	2JKGA	2JLIA	2JLQA	2LISA	2MCMA	2MEV1	2MHRA
2MPRA	2NLVA	2NLYA	2NMLA	2NMMA	2NN4A	2NNUA	2NO2A	2NOOA
2NPSD	2NPTA	2NPTB	2NQ2A	2NQWA	2NRHA	2NRJA	2NRKA	2NRQA
2NRRA	2NS0A	2NS6A	2NS9A	2NSAA	2NSCA	2NSZA	2NT0A	2NTXA
2NUHA	2NVOA	2NW8A	2NWAA	2NWFA	2NWLA	2NX2A	2NX9A	2NXFA
2NXOA	2NXPA	2NXVA	2NYIA	2NYKA	2NZ7A	2NZCA	2NZXA	2O0AA
2O0MA	2O0QA	2O1QA	2O2KA	2O2XA	2O30A	2O34A	2O38A	2O39C
2O3AA	2O3IA	2O3OA	2O4AA	2O4TA	2O4VA	2O5NA	2O5VA	2O62A
2O6KA	2O6PA	2O70A	2O71A	2O7GA	2O8GI	2O8PA	2O8SA	2O90A
2O9SA	2O9UX	2OA5A	2OAFA	2OB3A	2OB9A	2OBDA	2OBPA	2OD4A
2OD5A	2OD6A	2ODAA	2ODFA	2ODIA	2ODKA	2ODVA	2OEBA	2OEEA
2OEXA	2OEZA	2OF3A	2OFCA	2OFKA	2OG4A	2OGBA	2OGFA	2OGGA
2OH3A	2OHEA	2OHWA	2OITA	2OIZA	2OIZD	2OJ5A	2OKFA	2OKMA
2OKTA	2OKUA	2OL5A	2OLMA	2OLRA	2OLTA	2OMLA	2ONDA	2OOIA
2OOKA	2OPCA	2OPEA	2OPIA	2OPLA	2OQBA	2ORWA	2ORYA	2OSXA
2OTAA	2OTMA	2OU1A	2OU3A	2OU5A	2OU6A	2OV0A	2OVGA	2OVJA
2OWAA	2OWLA	2OX6A	2OX7A	2OXGB	2OXLA	2OXOA	2OY9A	2OYAA
2OYOA	2OYYA	2OYZA	2OZJA	2OZNB	2OZTA	2P02A	2P09A	2P0AA
2P0LA	2P0NA	2P0OA	2P0SA	2P0WA	2P11A	2P12A	2P14A	2P17A

2P1MB	2P22C	2P22D	2P26A	2P2SA	2P2UA	2P38A	2P3EA	2P3PA
2P3XA	2P3YA	2P4FA	2P4GA	2P51A	2P53A	2P58A	2P58C	2P5KA
2P61A	2P62A	2P63A	2P65A	2P67A	2P6VA	2P6WA	2P7VA	2P8GA
2P8IA	2P97A	2P9BA	2P9WA	2P9XA	2PA7A	2PAGA	2PBEA	2PCSA
2PD0A	2PD2A	2PFCA	2PFIA	2PFTA	2PFZA	2PGCA	2PGEA	2PGNA
2PGSA	2PH0A	2PH5A	2PH7A	2PHNA	2PHPA	2PIEA	2PIFA	2PIHA
2PJPA	2PJUA	2PKEA	2PKHA	2PM7A	2PMAA	2PMIB	2PNDA	2PNEA
2PNLA	2PNQA	2PNWA	2POFA	2PORA	2PP6A	2PPQA	2PQ7A	2PQ8A
2PQXA	2PR7A	2PRSA	2PRVA	2PRXA	2PS1A	2PS5A	2PSBA	2PSTX
2PT7G	2PTRA	2PV2A	2PV4A	2PVQA	2PW0A	2PW6A	2PW9A	2PWWA
2PXXA	2PY5A	2PYQA	2PYXA	2Q00A	2Q01A	2Q03A	2Q04A	2Q07A
2Q0SA	2Q0XA	2Q0ZX	2Q12A	2Q22A	2Q2IA	2Q30A	2Q35A	2Q3FA
2Q3QA	2Q3SA	2Q3TA	2Q40A	2Q43A	2Q48A	2Q4KA	2Q4MA	2Q4OA
2Q4XA	2Q4ZA	2Q5CA	2Q66A	2Q6KA	2Q6QA	2Q7AA	2Q7SA	2Q82A
2Q83A	2Q88A	2Q8KA	2Q8PA	2Q9KA	2Q9RA	2Q9UA	2QAPA	2QASA
2QB7A	2QCPX	2QDJA	2QDLA	2QDRA	2QE8A	2QEEA	2QF4A	2QFAB
2QFEA	2QGMA	2QGOA	2QGQA	2QGUA	2QGVA	2QGYA	2QH9A	2QHFA
2QHPA	2QHQA	2QIBA	2QIHA	2QIPA	2QJLA	2QJVA	2QJZA	2QKDA
2QKHA	2QKLB	2QKPA	2QKVA	2QL3A	2QL8A	2QLCA	2QMAA	2QMCB
2QMLA	2QMXA	2QN6B	2QNGA	2QNKA	2QNLA	2QNUA	2QOLA	2QP2A
2QPXA	2QQ4A	2QR4A	2QRLA	2QRUA	2QSBA	2QSDA	2QSFX	2QSIA
2QSKA	2QSQA	2QSVA	2QSWA	2QSXA	2QTQA	2QTSA	2QU7A	2QU8A
2QUFA	2QUOA	2QUPA	2QV3A	2QV5A	2QV6A	2QW5A	2QWWA	2QX5A
2QXFA	2QYAA	2QYBA	2QYFB	2QYWA	2QZBA	2QZCA	2QZIA	2QZQA
2R01A	2R0HA	2R0XA	2R16A	2R17C	2R19A	2R1FA	2R2AA	2R2CA
2R2YA	2R2ZA	2R31A	2R39A	2R3SA	2R41A	2R4FA	2R4GA	2R4IA
2R4QA	2R51A	2R5OA	2R5SA	2R5UA	2R6JA	2R6ZA	2R751	2R78A
2R7DA	2R7GA	2R85A	2R8EA	2R91A	2R9FA	2R9IA	2RA8A	2RA9A
2RAAA	2RBBA	2RBDA	2RBGA	2RBKA	2RC3A	2RD7A	2RDCA	2RDEA
2RDGA	2RDPA	2RDQA	2REUA	2REXB	2RFFA	2RFQA	2RG4A	2RG8A
2RGQA	2RGTA	2RH0A	2RH2A	2RH3A	2RHFA	2RHMA	2RHQB	2RHSA
2RIHA	2RILA	2RINA	2RIQA	2RJ2A	2RJIA	2RJNA	2RJZA	2RK9A
2RKHA	2RKLA	2RKNA	2RKVA	2RL8A	2RLDA	2SAKA	2SPCA	2TNFA
2TPSA	2TRCP	2UU8A	2UURA	2UUYB	2UUZA	2UVKA	2UWJE	2UXEA
2UXQA	2UY1A	2UYOA	2UYTA	2UZ1A	2UZCA	2V05A	2V0PA	2V0XA
2V14A	2V1MA	2V1YB	2V2GA	2V33A	2V3GA	2V3IA	2V3MA	2V3SA
2V4XA	2V66B	2V6GA	2V6VA	2V6YA	2V73A	2V75A	2V76A	2V79A
2V7FA	2V7KA	2V89A	2V8FA	2V8IA	2V8QA	2V8QB	2V8TA	2V9KA
2V9LA	2V9PA	2V9VA	2VA0A	2VAKA	2VBKA	2VBUA	2VC8A	2VCHA
2VCLA	2VDFA	2VDJA	2VDWA	2VDWB	2VE3A	2VE8A	2VEQA	2VEZA
2VFKA	2VFOA	2VFRA	2VFXA	2VGLS	2VGNA	2VH1A	2VH3A	2VHAA
2VHHA	2VK2A	2VK8A	2VKJA	2VLAA	2VLDA	2VLGA	2VLQA	2VLQB
2VN6A	2VNGA	2VO8A	2VOKA	2VOVA	2VPNA	2VPTA	2VPVA	2VPZC
2VQ2A	2VQCA	2VQGA	2VQPA	2VRSA	2VS7A	2VSGA	2VSMB	2VSOE

2VSYA	2VTWA	2VTYA	2VVEA	2VVFA	2VVPA	2VWAA	2VWSA	2VX8A
2VXBA	2VXGA	2VXNA	2VXPA	2VXXA	2VXZA	2VY1A	2VY8A	2VZCA
2VZPA	2W07B	2W0GA	2W15A	2W1JA	2W1RA	2W2GA	2W2RA	2W2SA
2W2XD	2W31A	2W39A	2W3QA	2W3XA	2W3YA	2W45A	2W4SA	2W4YA
2W50A	2W56A	2W5QA	2W61A	2W68A	2W6AA	2W7NA	2W7VA	2W7ZA
2W82A	2W83C	2W8MA	2W8TA	2W8XA	2W9JA	2W9YA	2WAAA	2WAWA
2WB0X	2WB7A	2WBIA	2WBNA	2WBTA	2WCRA	2WCWA	2WD5B	2WDCA
2WDQC	2WDQD	2WDSA	2WE3A	2WE5A	2WF7A	2WFIA	2WFOA	2WFPA
2WFWA	2WG8A	2WH6A	2WH7A	2WI8A	2WJ5A	2WJ9A	2WJNC	2WJRA
2WJVD	2WK1A	2WKDA	2WLRA	2WLUA	2WLVA	2WNFA	2WNKA	2WNPF
2WOLA	2WOYA	2WP0C	2WP7A	2WQ4A	2WQFA	2WQKA	2WSDA	2WT7B
2WTEA	2WTPA	2WUJA	2WUQA	2WURA	2WUSR	2WUXA	2WVQA	2WW5A
2WWXB	2WY3B	2WY4A	2WY8Q	2WYAA	2WZ1A	2WZBA	2WZKA	2WZOA
2WZPR	2WZR3	2X0DA	2X0QA	2X1DA	2X1QA	2X27X	2X29A	2X2SA
2X2UA	2X3DA	2X3GA	2X3JA	2X3MA	2X46A	2X49A	2X4JA	2X4KA
2X4LA	2X55A	2X5CA	2X5FA	2X5HA	2X5NA	2X5OA	2X5QA	2X5RA
2X5XA	2X5YA	2X65A	2X6NA	2X6WA	2X7IA	2X7QA	2X9KA	2X9ZA
2XBGA	2XC8A	2XCBA	2XD7A	2XDGA	2XDHA	2XDJA	2XEDA	2XEPA
2XESA	2XETA	2XEUA	2XFGB	2XFRA	2XFVA	2XG5B	2XGFA	2XGRA
2XHAA	2XHFA	2XHGA	2XI7A	2XIOA	2XJPA	2XKOC	2XLTA	2XMIA
2XMJA	2XN6A	2XOCA	2XODA	2XOMA	2XOVA	2XPPA	2XQHA	2XQQA
2XQUA	2XQXA	2XRHA	2XSEA	2XSKA	2XT2A	2XTCA	2XTPA	2XTSA
2XU0A	2XU3A	2XU6A	2XU8A	2XUAA	2XUBA	2XUVA	2XVYA	2XWSA
2XWVA	2XXNA	2XXPA	2XYKA	2XYQA	2XZ2A	2XZ9A	2XZEA	2XZIA
2Y0OA	2Y1BA	2Y1EA	2Y1HA	2Y24A	2Y26A	2Y3MA	2Y44A	2Y48B
2Y4YA	2Y53A	2Y5PA	2Y6UA	2Y6XA	2Y71A	2Y7PA	2Y8DA	2Y8GA
2Y8NB	2Y9MB	2Y9UA	2Y9WA	2Y9WC	2YANA	2YAVA	2YC2A	2YC3A
2YEQA	2YEVC	2YF2A	2YFRA	2YFUA	2YFVC	2YG9A	2YGBA	2YGGA
2YGKA	2YGNA	2YGUA	2YH5A	2YH6A	2YHAA	2YHCA	2YIMA	2YIZA
2YJGA	2YK4A	2YKFA	2YKTA	2YKZA	2YLEA	2YLMA	2YLNA	2YMAA
2YMOA	2YN0A	2YN5A	2YNAA	2YNKA	2YOGA	2YOPA	2YPVA	2YQ2A
2YV4A	2YVIA	2YVTA	2YWIA	2YX0A	2YXNA	2YXYA	2YY3A	2YYKA
2YYYA	2YZSA	2YZTA	2YZYA	2Z07A	2Z08A	2Z0BA	2Z0DA	2Z0JA
2Z0QA	2Z0RA	2Z0TA	2Z0XA	2Z14A	2Z1CA	2Z26A	2Z2NA	2Z30B
2Z3QA	2Z3XA	2Z51A	2Z5BA	2Z5BB	2Z5EA	2Z5WA	2Z6OA	2Z6RA
2Z72A	2Z84A	2Z86A	2Z8FA	2Z98A	2Z9WA	2ZA4B	2ZAHA	2ZB4A
2ZB9A	2ZBLA	2ZC3C	2ZCAA	2ZCMA	2ZD7A	2ZDIC	2ZDJA	2ZE7A
2ZEXA	2ZF9A	2ZFDB	2ZFGA	2ZFYA	2ZFZA	2ZGIA	2ZGYA	2ZHJA
2ZHPA	2ZI0A	2ZIHA	2ZIVB	2ZK9X	2ZKOA	2ZKZA	2ZMEB	2ZMEC
2ZNRA	2ZOSA	2ZOUA	2ZPMA	2ZPTX	2ZQ5A	2ZQKC	2ZQOA	2ZSIB
2ZSJA	2ZT5A	2ZTBA	2ZUXA	2ZW2A	2ZX2A	2ZXEB	2ZXXC	2ZYZB
2ZZ8A	2ZZJA	3A02A	3A07A	3A09A	3A0SA	3A0YA	3A16A	3A1FA
3A1JA	3A1JB	3A1JC	3A1PB	3A1YA	3A2EA	3A2ZA	3A35A	3A4CA
3A57A	3A5FA	3A5PA	3A5YA	3A6FA	3A6RA	3A72A	3A77A	3A7OA

3A8GA	3A8GB	3A8RA	3A98B	3A9FA	3A9IA	3A9LA	3A9SA	3AA0A
3AA0B	3AAFA	3ABDA	3ABHA	3ABQA	3ABQB	3ACHA	3ACXA	3ADGA
3ADYA	3AEHA	3AEIA	3AFCA	3AFOA	3AG3A	3AG3B	3AG3C	3AG3D
3AG3E	3AG3F	3AG3G	3AG3H	3AG3I	3AG3J	3AG7A	3AGCA	3AGKA
3AGNA	3AGYA	3AHNA	3AIAA	3AIHA	3AJ1A	3AJ7A	3AJDA	3AJFA
3AJIB	3AJWA	3AKJA	3AKSA	3AL2A	3ALRA	3AMRA	3AOFA	3AONA
3AONB	3AOTA	3AOWA	3APAA	3AQ2A	3AQBA	3AQEA	3AQOA	3AS8A
3ASLA	3ATSA	3AV3A	3AWUA	3AX1A	3AX2A	3AXDA	3AXGA	3AY5A
3AYHA	3AYHB	3B0DC	3B0XA	3B33A	3B47A	3B49A	3B4NA	3B4QA
3B4UA	3B5EA	3B5MA	3B5OA	3B64A	3B6EA	3B6HA	3B79A	3B7CA
3B7FA	3B85A	3B8FA	3B8OA	3B9OA	3B9PA	3B9TA	3B9WA	3BA3A
3BB0A	3BC1B	3BCYA	3BCZA	3BDUA	3BE3A	3BEDA	3BF7A	3BFMA
3BFQG	3BG1B	3BGEA	3BGYA	3BH0A	3BH1A	3BH4A	3BH7B	3BHDA
3BHPA	3BHWA	3BIDA	3BIQA	3BIYA	3BJDA	3BJEA	3BJNA	3BJQA
3BK3C	3BL9A	3BM1A	3BM3A	3BMAA	3BMZA	3BN0A	3BN3B	3BNVA
3BO6A	3BODA	3BOEA	3BOGA	3BONA	3BOWC	3BPJA	3BPKA	3BPVA
3BQ9A	3BQAA	3BQKA	3BQOA	3BQPA	3BQWA	3BQXA	3BRCA	3BRFA
3BRKX	3BRVB	3BS0A	3BS4A	3BS6A	3BS7A	3BSOA	3BT3A	3BT4A
3BT5A	3BTPA	3BUTA	3BUUA	3BUXB	3BV8A	3BVOA	3BWHA	3BWSA
3BWUD	3BWUF	3BWWA	3BWZA	3BXJA	3BXPA	3BXUA	3BXWA	3BY7A
3BY8A	3BY9A	3BYPA	3BYQA	3BYWA	3BZ6A	3BZMA	3BZWA	3C02A
3C0DA	3C0FB	3C0TA	3C0WA	3C18A	3C19A	3C1QA	3C25A	3C26A
3C2EA	3C2QA	3C2UA	3C37A	3C4RA	3C4SA	3C5KA	3C5NA	3C5PA
3C6AA	3C6FA	3C6KA	3C8CA	3C8GA	3C8IA	3C8LA	3C8OA	3C8WA
3C9FA	3C9HA	3CA7A	3CA8A	3CANA	3CBNA	3CBPA	3CBZA	3CCDA
3CCYA	3CDDA	3CE9A	3CEBA	3CECA	3CETA	3CEUA	3CEXA	3CG6A
3CGXA	3CHBD	3CHHA	3CHJA	3CHMA	3CI0I	3CI0K	3CI3A	3CIJA
3CIMA	3CINA	3CISA	3CITA	3CJDA	3CJLA	3CJPA	3CJSA	3CJSB
3CJXA	3CJYA	3CKCA	3CKDA	3CKJA	3CKKA	3CKMA	3CL5A	3CL6A
3CLJA	3CLMA	3CLQA	3CLWA	3CM1A	3CMNA	3CNBA	3CNEA	3CNIA
3CNVA	3CNYA	3COQA	3COVA	3CP0A	3CP3A	3CP7A	3CPQA	3CPTA
3CPTB	3CQ1A	3CQ5A	3CQCA	3CQCB	3CQXC	3CQYA	3CR3A	3CRJA
3CRKC	3CRYA	3CSVA	3CSXA	3CT5A	3CT6A	3CT9A	3CTPA	3CTRA
3CTWB	3CU9A	3CUZA	3CVEA	3CVOA	3CWFA	3CWNA	3CWRA	3CWXA
3CX2A	3CX4A	3CX5A	3CX5B	3CX5C	3CX5F	3CX5G	3CX5H	3CX5I
3CXBA	3CXJA	3CXNA	3CZ1A	3CZ8A	3CZPA	3CZXA	3D00A	3D0FA
3D0JA	3D19A	3D1BA	3D1KA	3D1PA	3D1RA	3D2OA	3D2QA	3D31C
3D32A	3D33A	3D34A	3D37A	3D3BA	3D3BJ	3D3KA	3D3SA	3D4EA
3D4UB	3D55A	3D6IA	3D6NB	3D6RA	3D6WA	3D7AA	3D7IA	3D8LA
3D8PA	3D8UA	3D9XA	3DA0A	3DA1A	3DA5A	3DA7A	3DADA	3DANA
3DB2A	3DB7A	3DBYA	3DCMX	3DCPA	3DCZA	3DD7A	3DDCB	3DDEA
3DDQB	3DDVA	3DEEA	3DEFA	3DELB	3DF6A	3DFEA	3DFFA	3DFGA
3DFUA	3DG6A	3DGPA	3DGPB	3DH4A	3DHAA	3DHXA	3DI2A	3DI5A
3DJEA	3DJLA	3DJMA	3DKAA	3DKRA	3DKXA	3DLCA	3DM8A	3DMBA

3DMLA	3DMNA	3DMYA	3DN7A	3DNHA	3DNJA	3DNSA	3DNXA	3DO6A
3DO9A	3DOAA	3DOEB	3DORA	3DOUA	3DPTA	3DQYA	3DR2A	3DR5A
3DR7A	3DRFA	3DS4A	3DSBA	3DSMA	3DSOA	3DSSA	3DT5A	3DTDA
3DTZA	3DUPA	3DUZA	3DV8A	3DVOA	3DWOX	3DX5A	3DXEA	3DXLA
3DXPA	3DXRA	3DXRB	3DYJA	3DZAA	3DZMA	3E0EA	3E0JB	3E0RA
3E0SA	3E0ZA	3E11A	3E1EA	3E1IA	3E2OA	3E3VA	3E3XA	3E4BA
3E4GA	3E4VA	3E4WA	3E59A	3E5TA	3E61A	3E6CC	3E7KA	3E7LA
3E8LC	3E8OA	3E8TA	3E8VA	3E99A	3E9KA	3E9VA	3EA6A	3EAFA
3EB8A	3EBEA	3EBWA	3EBYA	3EC3A	3ECFA	3EDOA	3EDPA	3EE4A
3EEAA	3EEBA	3EEHA	3EEQA	3EERA	3EF8A	3EFGA	3EFZA	3EGAA
3EGRA	3EGWC	3EHCA	3EI3B	3EIKA	3EIPA	3EJ9B	3EJKA	3EKIA
3EL6A	3ELEA	3ELFA	3ELIA	3ELKA	3EMFA	3EMIA	3ENCA	3EO6A
3EO7A	3EOIA	3EOJA	3EOQA	3EPSA	3EQXA	3ER6A	3ER7A	3ER9B
3ERMA	3ERVA	3ESLA	3ESMA	3ESSA	3ETJA	3ETOA	3ETVA	3ETZA
3EUHA	3EUHC	3EURA	3EVFA	3EVIA	3EVYA	3EWDA	3EXZA	3EYEA
3EZ0A	3EZIA	3F0DA	3F0PA	3F14A	3F1IH	3F1LA	3F1PB	3F1TA
3F2EA	3F2ZA	3F3KA	3F40A	3F4LA	3F4MA	3F62A	3F6CA	3F6VA
3F6YA	3F75P	3F79A	3F7EA	3F85A	3F8MA	3F8TA	3F95A	3F9SA
3F9XA	3FANA	3FAUA	3FAYA	3FBIA	3FBIB	3FBLA	3FBQA	3FBUA
3FBZA	3FCGA	3FCMA	3FCNA	3FD3A	3FD4A	3FD5A	3FD9A	3FDEA
3FDHA	3FDIA	3FDJA	3FDQA	3FDSC	3FEGA	3FETA	3FF1A	3FF5A
3FFRA	3FFVA	3FFYA	3FGAB	3FGHA	3FGRA	3FGRB	3FGVA	3FGXA
3FGYA	3FH3A	3FHVA	3FIAA	3FIDA	3FJ1A	3FJUB	3FJVA	3FK8A
3FKAA	3FKCA	3FKEA	3FKRA	3FLDA	3FLOA	3FLOB	3FLPA	3FM2A
3FM5A	3FMYA	3FN5A	3FNCA	3FNIA	3FO3A	3FO5A	3FO8D	3FOTA
3FOVA	3FPCA	3FPPA	3FPRA	3FQ8A	3FQMA	3FRHA	3FRNA	3FS3A
3FSOA	3FSSA	3FSTA	3FT1A	3FTJA	3FUCA	3FUTA	3FV9A	3FW9A
3FWBB	3FWYA	3FWZA	3FX3A	3FX7A	3FXDB	3FYBA	3FYMA	3FZ2A
3FZEA	3FZXA	3G0MA	3G16A	3G1JA	3G1PA	3G21A	3G23A	3G27A
3G2BA	3G2EA	3G36A	3G3KA	3G3SA	3G3TA	3G40A	3G4EA	3G4NA
3G5BA	3G5SA	3G5TA	3G67A	3G6EI	3G6EY	3G7PA	3G7UA	3G85A
3G8QA	3G8YA	3G91A	3G98A	3G9MA	3GA4A	3GA8A	3GAEA	3GB5A
3GBGA	3GBWA	3GD0A	3GDMA	3GDWA	3GE3A	3GE3C	3GE3E	3GETA
3GF6A	3GFAA	3GFJA	3GG7A	3GGNA	3GGYA	3GHAA	3GHFA	3GI7A
3GIAA	3GIUA	3GIWA	3GJYA	3GKEA	3GKJA	3GKMA	3GKRA	3GKUA
3GMGA	3GMIA	3GMOA	3GMXA	3GN3A	3GN6A	3GNAA	3GNEA	3GNFB
3GNLA	3GNZP	3GO5A	3GO9A	3GOCA	3GODA	3GOEA	3GOHA	3GONA
3GOZA	3GP4A	3GP6A	3GPVA	3GQHA	3GQQA	3GQXA	3GR3A	3GR4A
3GR5A	3GRAA	3GREA	3GS9A	3GTZA	3GUDA	3GV0A	3GVAA	3GVEA
3GVOA	3GVZA	3GW4A	3GWBA	3GWIA	3GWLA	3GWQA	3GWRA	3GXHA
3GXQA	3GXVA	3GYCA	3GZAA	3GZBA	3GZRA	3H05A	3H0DA	3H0NA
3H0UA	3H16A	3H1DA	3H20A	3H2ZA	3H35A	3H36A	3H38A	3H3IA
3H3LA	3H4CA	3H4RA	3H4SE	3H4TA	3H4XA	3H5LA	3H5TA	3H63A
3H6EA	3H6JA	3H6PC	3H6QA	3H6RA	3H74A	3H79A	3H7CX	3H7HB

3H7LA	3H87A	3H87C	3H8DA	3H8GA	3H8TA	3H8ZA	3H9CA	3H9MA
3H9PA	3H9WA	3HA4A	3HBMA	3HBXA	3HBZA	3HC7A	3HCYA	3HFHA
3HFIA	3HFOA	3HFTA	3HFWA	3HG9A	3HGTA	3HH1A	3HHWA	3HHWK
3HI0A	3HI2B	3HIDA	3HIEA	3HJ4A	3HJHA	3HKLA	3HKWA	3HL1A
3HL2A	3HL6A	3HLSA	3HLUA	3HLXA	3HLZA	3HM4A	3HMSA	3HN0A
3HN2A	3HNOA	3HNYM	3HO6A	3HO7A	3HOIA	3HP0A	3HP4A	3HPCX
3HPFA	3HQ1A	3HQFA	3HR0A	3HR6A	3HRDC	3HRGA	3HRLA	3HRQA
3HRZB	3HSAA	3HSIA	3HSLX	3HSUA	3HSYA	3HTKA	3HTKB	3HTRA
3HTSB	3HTUA	3HTYA	3HUFA	3HUGA	3HUGB	3HUUA	3HV8A	3HVAA
3HVWA	3HWUA	3HWWA	3HX3A	3HX8A	3HX9A	3HXJA	3HXLA	3HY0A
3HYIA	3HYNA	3HYWA	3HZ7A	3HZ8A	3HZBA	3HZPA	3HZSA	3I00A
3I0PA	3I0ZA	3I10A	3I16A	3I18A	3I1AA	3I2DA	3I2VA	3I33A
3I3VA	3I45A	3I4GA	3I4OA	3I4UA	3I4ZA	3I53A	3I57A	3I5QA
3I5TA	3I6XA	3I71A	3I76A	3I7AA	3I7MA	3I84A	3I94A	3I9YA
3IABA	3IABB	3IB5A	3IBWA	3IC8A	3ID1A	3IDBB	3IDFA	3IDWA
3IE4A	3IEEA	3IEYB	3IFRA	3IFUA	3IG2A	3IG9A	3IGHX	3IGMA
3IGQA	3IGZB	3IH6A	3II2A	3IISM	3IIXA	3IJ6A	3IJDA	3IJLA
3IJMA	3IJWA	3IK7A	3IKBA	3IKWA	3ILHA	3ILSA	3ILVA	3ILWA
3IM3A	3IM6A	3IMKA	3INGA	3IO5A	3IOHA	3IOXA	3IP0A	3IP3A
3IP4C	3IPFA	3IPJA	3IQWA	3IRBA	3IRPX	3IRVA	3IS6A	3ISMA
3ISMC	3ISQA	3ISYA	3IT3A	3IT4A	3IT4B	3IT5A	3IT8D	3ITFA
3ITQA	3IU0A	3IU6A	3IUKA	3IUOA	3IUPA	3IUSA	3IUVA	3IUWA
3IV0A	3IV1A	3IV7A	3IVVA	3IWFA	3IX0A	3IX3A	3IXLA	3IXSA
3JQ0A	3JQ1A	3JQOA	3JRNA	3JRTA	3JRVA	3JSRA	3JSYA	3JTMA
3JTNA	3JTXA	3JTZA	3JU2A	3JUDA	3JUIA	3JUMA	3JV1A	3JVOA
3JX9A	3JXPA	3JXYA	3JY6A	3JYBA	3JYOA	3JYZA	3JZ3A	3K05A
3K0ZA	3K1HA	3K1TA	3K1UA	3K1ZA	3K25A	3K26A	3K29A	3K2IA
3K2OA	3K2YA	3K2ZA	3K3CA	3K3VA	3K40A	3K4IA	3K4TA	3K5JA
3K63A	3K67A	3K69A	3K6MA	3K6OA	3K6QA	3K6YA	3K7CA	3K7IB
3K85A	3K8GA	3K8PC	3K8PD	3K8RA	3K8UA	3K93A	3K94A	3K9TA
3KA5A	3KAEA	3KB2A	3KB9A	3KBGA	3KBRA	3KD3A	3KD4A	3KDEC
3KDGA	3KDRA	3KE2A	3KE3A	3KE7A	3KEPA	3KEVA	3KEYA	3KF6A
3KF6B	3KF8B	3KFFA	3KFOA	3KFWX	3KG7A	3KG9A	3KGKA	3KGWA
3KH1A	3KHKA	3KIZA	3KJXA	3KK7A	3KKFA	3KKGA	3KLJA	3KLQA
3KLUA	3KM5A	3KMIA	3KNYA	3KOGA	3KORA	3KP1E	3KP8A	3KQ0A
3KQ5A	3KS6A	3KS9A	3KSNA	3KSPA	3KT7A	3KTDA	3KTOA	3KUPA
3KUVA	3KV1A	3KVCA	3KVHA	3KW2A	3KWEA	3KWRA	3KXEA	3KXEC
3KXRA	3KXSA	3KXTA	3KXYA	3KXYT	3KY8A	3KYFA	3KYJA	3KYLA
3KYPA	3KYZA	3KZHA	3KZJA	3KZPA	3L00A	3L09A	3L0AA	3L0FA
3L15A	3L1LA	3L1WA	3L2CA	3L39A	3L42A	3L49A	3L4CA	3L4FA
3L51B	3L60A	3L6BA	3L6IA	3L7HA	3L81A	3L82B	3L8WA	3L9AX
3LAAA	3LAGA	3LATA	3LAXA	3LB2A	3LCZA	3LD1A	3LD7A	3LDCA
3LDQB	3LE4A	3LEDA	3LEQA	3LETA	3LEWA	3LFRA	3LFTA	3LGBA
3LGDA	3LH2S	3LHCA	3LHEA	3LHIA	3LHLA	3LHNA	3LHOA	3LHXA

3LICA	3LIDA	3LJBA	3LKEA	3LKMA	3LLBA	3LLKA	3LLOA	3LLPA
3LM2A	3LM3A	3LM4A	3LMBA	3LMFA	3LMZA	3LNLA	3LO8A	3LOGA
3LP5A	3LPHA	3LPZA	3LQ9A	3LQBA	3LQCA	3LQKA	3LRQA	3LRVA
3LS9A	3LSNA	3LSOA	3LTIA	3LUQA	3LURA	3LUUA	3LUYA	3LVKB
3LVUA	3LWTX	3LWXA	3LXRF	3LXZA	3LYBA	3LYEA	3LYGA	3LYHA
3LYSA	3LYWA	3LZQA	3M03A	3M07A	3M0AA	3M0MA	3M1CB	3M1TA
3M1XA	3M2TA	3M31A	3M33A	3M3PA	3M4IA	3M4RA	3M4WA	3M5QA
3M66A	3M6JA	3M6NA	3M6ZA	3M73A	3M7AA	3M7KA	3M7NA	3M7OA
3M89A	3M8JA	3M9QA	3M9VA	3MABA	3MAHA	3MAYA	3MAZA	3MB2B
3MBRX	3MC3A	3MC9A	3MCBA	3MCBB	3MCPA	3MCRA	3MCSA	3MCWA
3MCXA	3MD9A	3MDMA	3MDNA	3MDPA	3MDQA	3MDUA	3ME0B	3ME7A
3MEAA	3MEMA	3MFBA	3MFXA	3MGAA	3MHXA	3MI01	3MILA	3MJ0A
3MJGA	3MJOA	3MK4A	3MKCA	3MKHA	3MKOA	3MKRB	3MKZA	3ML3A
3MLNA	3MLQA	3MMHA	3MMPF	3MMYB	3MNFA	3MPKA	3MQ0A	3MQ1A
3MQDA	3MQZA	3MR0A	3MRUA	3MSHA	3MSOA	3MSQA	3MSWA	3MSXB
3MT0A	3MT1A	3MT5A	3MTKA	3MTSA	3MTVA	3MU7A	3MUJA	3MUQA
3MVAO	3MVCA	3MVNA	3MVSA	3MVUA	3MW8A	3MWCA	3MWDA	3MWXA
3MWZA	3MX7A	3MXNA	3MXNB	3MXOA	3MXZA	3MY2A	3MYDA	3MYFA
3MYOA	3MYXA	3MZ0A	3MZ2A	3MZKB	3N01A	3N08A	3N0AA	3N0WA
3N0XA	3N10A	3N17A	3N1EA	3N1MC	3N2WA	3N2ZB	3N4JA	3N4SA
3N54B	3N6TA	3N6XA	3N6YA	3N6ZA	3N70A	3N7XA	3N89A	3N8BA
3N91A	3N9YA	3NBCA	3NBIA	3NBMA	3NCEA	3NCTA	3ND1A	3NDQA
3NE5B	3NE8A	3NEKA	3NETA	3NFGA	3NFGB	3NFIA	3NFTA	3NG9A
3NGQA	3NGXA	3NHEA	3NI0A	3NJEA	3NJNA	3NK4A	3NKEA	3NKGA
3NKUA	3NMDA	3NN1A	3NNBA	3NNGA	3NO0A	3NO2A	3NO3A	3NO7A
3NOHA	3NOJA	3NOQA	3NPDA	3NPHB	3NPPA	3NQIA	3NQNA	3NQZA
3NR1A	3NR5A	3NREA	3NRFA	3NRHA	3NRLA	3NRSA	3NRWA	3NRXA
3NS4A	3NTXA	3NUFA	3NUQA	3NV0A	3NVOA	3NVSA	3NVWB	3NVXA
3NY3A	3NYBB	3NYMA	3NYWA	3NZLA	3O0GD	3O0LA	3O0YA	3O0ZA
3O10A	3O12A	3O1IC	3O27A	3O2TA	3O3MA	3O3MB	3O48A	3O4PA
3O5TA	3O5YA	3O65A	3O6QA	3O6UA	3O6ZA	3O7BA	3O7IA	3O83A
3O8MA	3O8QA	3O94A	3O9ZA	3OAEA	3OAJA	3OAMA	3OAOA	3OBEA
3OBHA	3OBLA	3OC8A	3OCUA	3OD1A	3OD8A	3ODMA	3ODNA	3OE3A
3OEPA	3OF4A	3OF6D	3OFGA	3OG6A	3OG6B	3OGNA	3OHEA	3OHGA
3OHSX	3OIOA	3OIQA	3OISA	3OIZA	3OJCA	3OKGA	3OKQA	3OKXA
3OKZA	3OLCX	3OM0A	3OMYA	3ON9A	3ONDA	3ONHA	3ONJA	3ONTA
3OO8A	3OOSA	3OOUA	3OOXA	3OP6A	3OQ4A	3OQIA	3OQQA	3OR1A
3ORJA	3ORUA	3OS4A	3OSEA	3OSTA	3OSVA	3OT1A	3OT2A	3OT9A
3OTDA	3OTNA	3OUNB	3OUVA	3OV5A	3OV8A	3OV9A	3OVBA	3OVKA
3OWRA	3OXNA	3OXPA	3OY2A	3OYOA	3OYVA	3OYZA	3P02A	3P06A
3P0KA	3P0UA	3P0WA	3P1VA	3P2CA	3P2EA	3P2HA	3P2TA	3P3CA
3P3DA	3P4GA	3P4HA	3P51A	3P5JB	3P5JC	3P6LA	3P8AA	3P8CF
3P9AA	3P9VA	3P9ZA	3PASA	3PB6X	3PC7A	3PCVA	3PD7A	3PDYA
3PE7A	3PE9A	3PESA	3PEVB	3PF0A	3PF6A	3PF7A	3PFEA	3PFGA

3PFTA	3PG6A	3PG7A	3PGUA	3PH9A	3PHXA	3PHXB	3PICA	3PIUA
3PIWA	3PJ0A	3PJPA	3PJVD	3PKZA	3PL0A	3PLTA	3PLWA	3PM2A
3PMCA	3PMEA	3PMGA	3PMSA	3PN3A	3PNRB	3PNXA	3POHA	3POJA
3POPA	3POWA	3PP2A	3PP5A	3PPLA	3PPMA	3PQHA	3PR6A	3PROC
3PSHA	3PT8B	3PU2A	3PU9A	3PUCA	3PUIA	3PV8A	3PVEA	3PVHA
3PVIA	3PVKA	3PVVA	3PVZA	3PXLA	3PYCA	3PYWA	3PZ6A	3PZDA
3PZSA	3Q0HA	3Q18A	3Q1CA	3Q1IA	3Q1NA	3Q20A	3Q2IA	3Q2UA
3Q3MB	3Q3YA	3Q46A	3Q49B	3Q4HA	3Q4OA	3Q63A	3Q64A	3Q6AA
3Q6BA	3Q6CA	3Q6KA	3Q7CA	3Q7RA	3Q87B	3Q8DA	3QBTB	3QC5X
3QC7A	3QD7X	3QDKA	3QE7A	3QECA	3QEEA	3QEKA	3QF2A	3QF7A
3QFLA	3QFMA	3QFWA	3QGUA	3QH6A	3QH9A	3QHBA	3QHOA	3QHQA
3QI7A	3QJGA	3QK7A	3QL6A	3QL9A	3QM9A	3QMDA	3QMLC	3QNSA
3QOWA	3QP4A	3QP9A	3QPAA	3QQ2A	3QQYA	3QQZA	3QR7A	3QRAA
3QS2A	3QSJA	3QSQA	3QSZA	3QTAA	3QTHA	3QTMA	3QU3A	3QU5A
3QVLA	3QVPA	3QW9A	3QWBA	3QWEA	3QWGA	3QWLA	3QWMA	3QWNA
3QWUA	3QX1A	3QXFA	3QXZA	3QY3A	3QY7A	3QY9A	3QYEA	3QZBA
3QZMA	3QZXA	3R07C	3R15A	3R2CA	3R2QA	3R45C	3R4IA	3R4RA
3R4VA	3R4ZA	3R5TA	3R5ZA	3R6AA	3R6DA	3R6UA	3R72A	3R84A
3R84B	3R87A	3R8JA	3R90A	3R9FA	3RAUA	3RBYA	3RC9A	3RCOA
3RD7A	3RETA	3RF0A	3RF3A	3RFAA	3RFEA	3RG8A	3RGCA	3RGQA
3RHTA	3RIPA	3RJ2X	3RJPA	3RJVA	3RK1A	3RK6A	3RKCA	3RKGA
3RKLA	3RKOA	3RKOC	3RKOD	3RKOF	3RKOG	3RL5A	3RLFG	3RLKA
3RLOA	3RLSA	3RM3A	3RMHA	3RMQA	3RNLA	3RNVA	3RO3A	3ROBA
3RONA	3RPCA	3RPDA	3RPFA	3RPJA	3RPPA	3RQ9A	3RQAA	3RQTA
3RQWA	3RRIA	3RRKA	3RSNA	3RT2A	3RT3C	3RTLA	3RUIA	3RVAA
3RWNA	3RX6A	3RX9A	3RXYA	3RY3A	3RZNA	3S0AA	3S0YA	3S25A
3S2JA	3S2KC	3S2RA	3S44A	3S5TA	3S5WA	3S63A	3S64A	3S6EA
3S6FA	3S6N2	3S6PA	3S8GA	3S8IA	3S8MA	3S8SA	3S98A	3S9JA
3SB4A	3SBMA	3SBTB	3SC0A	3SC7X	3SCYA	3SEEA	3SEOA	3SG0A
3SGGA	3SGHA	3SGWA	3SHGA	3SHGB	3SHPA	3SIGA	3SIMA	3SJ5A
3SJAC	3SJMA	3SJRA	3SK7A	3SK9A	3SKQA	3SKVA	3SL9A	3SLRA
3SMPA	3SMZA	3SNKA	3SNOA	3SO6A	3SOJA	3SOKA	3SONA	3SOYA
3SPEA	3SSCA	3SU6A	3SUKA	3SUMA	3SWHA	3SWKA	3SWOA	3SXOA
3SXUA	3SXUB	3SY1A	3SY6A	3SZYA	3T0HA	3T2CA	3T33A	3T3LA
3T3OA	3T47A	3T4LA	3T4RA	3T5NA	3T5VA	3T5XA	3T61A	3T6AA
3T6GB	3T6OA	3T6SA	3T7DA	3T7KA	3T7LA	3T7ZA	3T8JA	3T8KA
3T92A	3T94A	3T97A	3T97B	3T9OA	3T9WA	3TBDA	3TBIA	3TC3A
3TC8A	3TCMA	3TCVA	3TDGA	3TDQA	3TDSA	3TDUC	3TDWA	3TE6A
3TE8A	3TEBA	3TEEA	3TEKA	3TEQA	3TEUA	3TEWA	3TFGA	3TFJA
3TG2A	3TG9A	3THFA	3THGA	3TIPA	3TIWA	3TJ1A	3TJMA	3TJYA
3TJZB	3TJZC	3TL8B	3TLQA	3TM8A	3TMGA	3TN2A	3TNYA	3TO3A
3TOSA	3TOWA	3TPDA	3TQ7B	3TR2A	3TRKA	3TRTA	3TS3A	3TS9A
3TSIA	3TT9A	3TU8A	3TUFA	3TUIA	3TUOA	3TUTA	3TV0A	3TVJA
3TVKA	3TVQA	3TW5A	3TW8A	3TWDA	3TWLA	3TX3A	3TX8A	3TXNA

3TXSA	3TZGA	3TZYA	3U02A	3U07A	3U0HA	3U0RA	3U0VA	3U12A
3U1DA	3U1UA	3U21A	3U24A	3U2GA	3U2RA	3U2UA	3U3LC	3U49A
3U4GA	3U4KA	3U4VA	3U4YA	3U4ZA	3U50C	3U52C	3U52E	3U5SA
3U5VA	3U5WA	3U64A	3U65A	3U6GA	3U6XS	3U7QA	3U7QB	3U7ZA
3U8VA	3U97A	3U99A	3U9JA	3U9QA	3U9RB	3UA0A	3UAFA	3UANA
3UAUA	3UB1A	3UB2A	3UB6A	3UBYA	3UCSA	3UD1A	3UEBA	3UEJA
3UF6A	3UF7A	3UFBA	3UFEA	3UFIA	3UG9A	3UGOA	3UGUA	3UH8A
3UIDA	3UIPD	3UITA	3UJCA	3ULBA	3ULJA	3ULLA	3ULTA	3UMHA
3UMZA	3UOAB	3UP3A	3UP6A	3UPLA	3UPSA	3UR8A	3URRA	3URZA
3US3A	3USHA	3UT4A	3UUEA	3UUNA	3UUWA	3UV0A	3UV1A	3UWSA
3UWSB	3UXJA	3UZQB	3V0DA	3V0RA	3V2UA	3V33A	3V39A	3V3LA
3V42A	3V46A	3V47C	3V4KA	3V5CA	3V5UA	3V68A	3V69A	3V6IA
3V6IB	3V6OA	3V71A	3V7BA	3V7NA	3V85A	3V93A	3V96A	3V9OA
3V9RB	3V9WA	3VA9A	3VBCA	3VC8A	3VCFA	3VDJA	3VEAA	3VENA
3VG8A	3VGIA	3VGLA	3VGPA	3VHJA	3VHLA	3VHXB	3VIIA	3VIQA
3VIQB	3VJ9A	3VJFA	3VK5A	3VK6A	3VKWA	3VL1A	3VL9A	3VLAA
3VLDA	3VN3A	3VNRA	3VNYA	3VOQA	3VORA	3VP9A	3VPBA	3VPBE
3VPZA	3VQFA	3VQJA	3VR0A	3VRHA	3VS8A	3VSJB	3VU9A	3VU9B
3VUBA	3VUPA	3VUSA	3VV1A	3VVVA	3VWAA	3VWBA	3VWCA	3VWIA
3VWNX	3VX0A	3VX3A	3VXJA	3VXVA	3VYWA	3VZ6A	3VZ9B	3VZ9D
3VZHA	3VZXA	3W06A	3W07A	3W0EA	3W0FA	3W0KA	3W0OA	3W1EA
3W1OA	3W1ZA	3W20A	3W2WB	3W2ZA	3W36A	3W3WA	3W42A	3W4TA
3W54A	3W5HA	3W61A	3W6DA	3W6JA	3W6KB	3W6SA	3W8QA	3W9EA
3W9SA	3WA1A	3WARA	3WASA	3WAZA	3WCOA	3WCQA	3WCTB	3WDCA
3WDGB	3WDNA	3WDQA	3WE2A	3WE9A	3WFDB	3WG9A	3WGQA	3WGTA
3WGXA	3WH1A	3WH2A	3WHJA	3WHRA	3WHTB	3WHXB	3WI3A	3WI5A
3WISA	3WITA	3WIWA	3WJ9A	3WJDA	3WJPA	3WJTA	3WKGA	3WKRC
3WL4A	3WL8A	3WMIA	3WMTA	3WMVA	3WMWA	3WNDA	3WNZA	3WO6A
3WOEB	3WPUA	3WQCA	3WQMA	3WQTA	3WRBA	3WRYA	3WTDA	3WTTB
3WU2A	3WU2B	3WU2E	3WU2H	3WU2O	3WU2U	3WU2Z	3WURA	3WUZA
3WV4A	3WVQA	3WW9A	3WWLA	3WX4A	3WXFA	3WXMB	3WZSA	3ZBGA
3ZBOA	3ZC0A	3ZC4A	3ZCOA	3ZDBA	3ZDOA	3ZDSA	3ZE3A	3ZEUB
3ZF8A	3ZFIA	3ZFPA	3ZGHA	3ZH5A	3ZH9B	3ZHEA	3ZHIA	3ZIAA
3ZIDA	3ZIEA	3ZIGA	3ZIHA	3ZILA	3ZJ0A	3ZJAA	3ZJBA	3ZJEA
3ZK4A	3ZL8A	3ZLCA	3ZN3A	3ZN4A	3ZN6A	3ZNUA	3ZNVA	3ZOJA
3ZPJA	3ZPLA	3ZPNA	3ZPXA	3ZQOA	3ZQUA	3ZR8X	3ZRGA	3ZRXA
3ZSJA	3ZSUA	3ZT9A	3ZTAA	3ZTHA	3ZUCA	3ZUIA	3ZUZA	3ZVKA
3ZVKE	3ZW5A	3ZXCA	3ZXKA	3ZXNA	3ZY2A	3ZY7A	3ZYPA	3ZYTA
3ZZHA	3ZZOA	3ZZPA	3ZZYA	4A02A	4A0EA	4A1GA	4A1RA	4A20A
4A27A	4A29A	4A2BA	4A2VA	4A35A	4A37A	4A3PA	4A4JA	4A56A
4A5UA	4A5UB	4A5XA	4A69C	4A6QA	4A7UA	4A8JA	4A8JB	4A8JC
4A94C	4A9CA	4A9VA	4AB5A	4ABLA	4ABMA	4ABYA	4AC1X	4AC7A
4AC7B	4ACFA	4ACJA	4ACKA	4ACOA	4ACVA	4ADIA	4ADNA	4ADZA
4AE0A	4AE2A	4AE7A	4AEQA	4AF8A	4AFFA	4AFKA	4AFMA	4AG6A

4AGKA	4AIWA	4AJSA	4AJYC	4AJYV	4AKKA	4AKLA	4AKMA	4AKXA
4AL0A	4ALZA	4AMQA	4ANNA	4ANOA	4AO6A	4AP5A	4APOA	4AQNA
4AQOA	4AQRD	4AR9A	4ARTA	4ARUA	4AS2A	4ASCA	4ASMB	4AT7B
4ATEA	4ATGA	4ATHA	4ATMA	4AU1A	4AURA	4AVRA	4AVSA	4AWNA
4AXDA	4AXOA	4AY0A	4AY9A	4AYOA	4AZ6A	4B0FA	4B0MA	4B0TA
4B1MA	4B1YB	4B21A	4B28A	4B2FA	4B2OA	4B3BA	4B4CA	4B4DA
4B4YA	4B5OA	4B60A	4B62A	4B6HA	4B6XA	4B89A	4B8EA	4B8VA
4B8XA	4B9GA	4BAXA	4BB9A	4BC3A	4BEUA	4BFCA	4BFOA	4BG2A
4BG7A	4BGBA	4BGCA	4BGPA	4BH6A	4BH6I	4BHQA	4BHRA	4BHUA
4BI4A	4BJ0A	4BJ1A	4BJAA	4BJIA	4BJJA	4BJJB	4BJMA	4BJQA
4BJSA	4BJTA	4BJUA	4BJZA	4BK0A	4BK7A	4BKWA	4BL6A	4BLPA
4BLQA	4BLUA	4BMDA	4BMJA	4BN4A	4BNDA	4BOEA	4BOJA	4BOQA
4BOUA	4BPFA	4BPSA	4BPUB	4BPYA	4BPZA	4BQ6D	4BQNA	4BQYA
4BRCA	4BRYA	4BSPA	4BSVA	4BSXA	4BT7A	4BT9A	4BUGA	4BUOA
4BVNA	4BVQA	4BWCA	4BWDA	4BWRA	4BWZA	4BX8A	4BX9C	4BXMA
4BXOB	4BY6A	4BYZA	4BZ4A	4BZAA	4BZPA	4C0FA	4C0NA	4C0OC
4C0ZA	4C12A	4C1NK	4C1SA	4C1WA	4C1YA	4C24A	4C2M4	4C2MD
4C2MI	4C2OA	4C47A	4C5CA	4C5KA	4C5WA	4C69X	4C6AA	4C6EA
4C6SA	4C76A	4C7AA	4C7GA	4C8DA	4C93A	4C97A	4C9BB	4C9SA
4C9XA	4C9YA	4CA1A	4CADC	4CAHB	4CAYA	4CAYB	4CBCA	4CBEA
4CBHA	4CBPA	4CBUG	4CC2A	4CC9B	4CCKA	4CCVA	4CCWA	4CD5A
4CD8A	4CDJA	4CE8A	4CFIA	4CFPA	4CG1A	4CGOA	4CGSA	4CGUB
4CHDA	4CHEA	4CHIA	4CHMA	4CHSA	4CIHA	4CIIA	4CIJA	4CITA
4CJ0A	4CJ0B	4CJDA	4CL7A	4CLCA	4CLLA	4CLQA	4CLQB	4CMRA
4CNGA	4CNKA	4CNNA	4CO6A	4CO8A	4COFA	4COGA	4CPCA	4CQHA
4CRHA	4CRUA	4CRUB	4CRWB	4CS4A	4CS9A	4CSSA	4CT3A	4CU2A
4CU5A	4CUAA	4CV7A	4CVNE	4CVOA	4CVRA	4CW5A	4CXPA	4CY9A
4CYJE	4CZGA	4CZXA	4CZXB	4D04A	4D05A	4D0NB	4D0QA	4D1JA
4D2IA	4D3TA	4D5TA	4D6GA	4D6KA	4D6QA	4D7CA	4D8BA	4D8MA
4D9BA	4D9IA	4D9SA	4DA2A	4DAMA	4DBGB	4DCKA	4DCZA	4DD5A
4DDPA	4DEYA	4DFCB	4DGFA	4DGUA	4DHXA	4DI9A	4DIDB	4DJAA
4DJDD	4DK2A	4DKCA	4DKJA	4DKKA	4DKNA	4DKWA	4DLHA	4DLQA
4DM5A	4DMIA	4DMOA	4DMVA	4DNYA	4DO4A	4DOIA	4DOOA	4DOXA
4DPZX	4DQ6A	4DQ9A	4DQAA	4DQJA	4DQZA	4DRIB	4DSSA	4DT5A
4DUIA	4DUQA	4DV8A	4DVEA	4DVGB	4DVKA	4DW1A	4DWEA	4DWLA
4DWRA	4DXTA	4DYLA	4DYNA	4DYOA	4DYQA	4DZBA	4DZIA	4DZOA
4E0AA	4E0EA	4E0GA	4E1PA	4E1SA	4E29A	4E2XA	4E3EA	4E3XA
4E3YA	4E40A	4E45E	4E4WA	4E4WB	4E5VA	4E5XG	4E6KG	4E6NA
4E6SA	4E6WA	4E6ZA	4E72A	4E74A	4E8UA	4E9FA	4E9SA	4EA9A
4EACA	4EADA	4EAEA	4EATA	4EBGA	4ECFA	4EDPA	4EE6A	4EETB
4EFAE	4EFAG	4EFPA	4EGCB	4EGDA	4EGUA	4EGVA	4EHSA	4EHXA
4EI0A	4EICA	4EIJA	4EIRA	4EIUA	4EJQA	4EJRA	4EJYA	4EKFA
4EKXA	4EL6A	4EMNA	4EMOA	4EMTA	4ENEA	4ENFA	4EO0A	4EO7A
4EOZB	4EP4A	4EPZA	4EQ3A	4EQ8A	4EQAC	4EQBA	4EQLA	4EQQA

4ER8A	4ERCA	4ERNA	4ERYA	4ES8A	4ESFA	4ESMA	4ESQA	4ESUA
4ETNA	4ETRA	4EU0A	4EU9A	4EUOA	4EUYA	4EV1A	4EVUA	4EVWA
4EW5A	4EW7A	4EWCA	4EXKA	4EXOA	4EXQA	4EXTA	4EYSA	4EYZA
4F01A	4F06A	4F0QA	4F1VA	4F27A	4F2DA	4F2EA	4F2LA	4F2ME
4F3FC	4F4WA	4F52A	4F52E	4F55A	4F7GB	4F7HA	4F7UP	4F87A
4F8LA	4F9CB	4FA8A	4FA8E	4FBCA	4FBIA	4FBJA	4FBSA	4FBWA
4FC3E	4FC5A	4FCAA	4FCHA	4FDBA	4FDTA	4FDZA	4FEKA	4FFBC
4FGCA	4FGQA	4FGWA	4FHRA	4FHRB	4FKMA	4FMPA	4FN7A	4FO0A
4FP5D	4FPRA	4FQEA	4FQGA	4FQNA	4FTFA	4FTXA	4FUVA	4FVGA
4FVQA	4FWVA	4FX7A	4FXIA	4FXWB	4FYPA	4FZ4A	4FZLA	4FZQA
4FZSA	4G08A	4G0AA	4G0RA	4G0XA	4G10A	4G1IA	4G1OA	4G22A
4G26A	4G29A	4G2SA	4G38A	4G3HA	4G3NA	4G3OA	4G3VA	4G4KA
4G4SO	4G4SP	4G54A	4G55A	4G68A	4G6DB	4G6IA	4G6UA	4G6VA
4G79A	4G7NA	4G7XA	4G7XB	4G92A	4G97A	4G9EA	4G9PA	4GA2A
4GAIA	4GAXA	4GB5A	4GBMA	4GC0A	4GC3A	4GDZA	4GEHA	4GEIA
4GEYA	4GF3A	4GGJA	4GGVA	4GHKA	4GI3C	4GIOA	4GIPA	4GIWA
4GIZC	4GJ4A	4GJRA	4GJZA	4GKGA	4GKHA	4GLKA	4GMFA	4GMOA
4GMQA	4GMUA	4GN5A	4GNRA	4GNXB	4GOFA	4GOQA	4GOUA	4GQ4A
4GQZA	4GRNA	4GS1A	4GS3A	4GT8A	4GT9A	4GU5A	4GUCA	4GVBB
4GVFA	4GVQA	4GWBA	4GWGA	4GX7A	4GXBA	4GXWA	4GYOA	4GYTA
4GYVA	4GZ1A	4GZCA	4GZRA	4GZRB	4H0FA	4H14A	4H1BA	4H22A
4H2WA	4H3SA	4H3TA	4H3WA	4H40A	4H4DA	4H4GA	4H4NA	4H4VA
4H59A	4H5BA	4H5IA	4H5SA	4H61A	4H62Q	4H6CA	4H6QA	4H7LA
4H7WA	4H7YA	4H8EA	4H9NC	4HA7A	4HBQA	4HBSA	4HC9A	4HCEA
4HCJA	4HCSA	4HCWA	4HCZA	4HD5A	4HDDA	4HDEA	4HDOA	4HE6A
4HE7A	4HEIA	4HEOA	4HFQA	4HFVA	4HGXA	4HH3C	4HH8A	4HHJA
4HHOA	4HHVA	4HI0B	4HI4A	4HI6A	4HI7A	4HIKA	4HJ1A	4HKGA
4HKHA	4HKJD	4HL2A	4HL4A	4HLBA	4HLSA	4HMSA	4HN7A	4HNEA
4HNOA	4HOBA	4HPMA	4HPMB	4HQBA	4HQEA	4HR1A	4HR9A	4HRNC
4HRSA	4HRVA	4HS1A	4HS2A	4HSCX	4HSPA	4HSSA	4HSTB	4HT3B
4HT5A	4HTEA	4HTGA	4HU2A	4HUDA	4HUQS	4HUQT	4HV0A	4HVKA
4HVMA	4HW6A	4HWCA	4HWMA	4HWVA	4HY4A	4HYLA	4HYQA	4HZ9A
4HZ9B	4HZFA	4HZOA	4I0OA	4I0UA	4I0WA	4I0XA	4I0XB	4I16A
4I1KA	4I1LA	4I1OB	4I1SB	4I2AA	4I2OA	4I3MA	4I3YA	4I4NA
4I4OA	4I4TA	4I4TE	4I4TF	4I66A	4I68A	4I6JB	4I6MA	4I6MB
4I6MC	4I6MD	4I6XA	4I6YA	4I79A	4I82A	4I84A	4I86A	4I8IA
4I8OA	4I90A	4I93A	4I99C	4I9OA	4I9XA	4IA6A	4IABA	4IAJA
4IAOC	4IAUA	4IC3A	4IC4A	4IC9A	4ICGC	4ICVA	4ICWA	4ID2A
4IDHA	4IDOA	4IE5A	4IEFA	4IEJA	4IEUA	4IG1A	4IGIA	4IGKA
4IGVA	4IHQA	4IHZA	4II1A	4IIKA	4IILA	4IIYA	4IJNA	4IJYA
4IKCA	4IKDA	4IKGA	4IKNA	4IKVA	4IL7A	4ILLA	4IM7A	4IN0A
4INDA	4INOA	4INWA	4INZA	4IOXA	4IOYX	4IPIA	4IPUA	4IQBA
4IQNA	4IQZA	4IRFA	4IRVA	4ISBA	4ISVB	4IT6A	4ITJA	4ITQA
4ITRA	4IU3B	4IUHA	4IUJA	4IUMA	4IUPA	4IUSA	4IWBA	4IWNA

4IX1A	4IX3A	4IX7A	4IXJA	4IYAA	4IYJA	4IZ7B	4IZHA	4IZUA
4IZXA	4J05A	4J0WA	4J1PA	4J1QA	4J1VA	4J27A	4J2CA	4J2GA
4J2NA	4J32A	4J32B	4J37A	4J39A	4J42A	4J4HA	4J4ZA	4J5RA
4J6EA	4J6OA	4J7HA	4J7NA	4J7OA	4J7QA	4J8LA	4J8SA	4J91A
4J9YB	4JB7A	4JBUA	4JCCA	4JCYA	4JD9A	4JDEB	4JDNA	4JDUA
4JDXA	4JEMA	4JF5A	4JF8A	4JG9A	4JGIA	4JGLA	4JHCA	4JHDC
4JHKA	4JHLA	4JHMA	4JHTA	4JIFA	4JIUA	4JJAA	4JJOA	4JK8A
4JL5A	4JLEA	4JM1A	4JMDA	4JMFA	4JMQA	4JMUA	4JN3A	4JN7A
4JN9A	4JNHA	4JO0A	4JO7A	4JOBA	4JOIA	4JOIC	4JOQA	4JOXA
4JP0A	4JP6A	4JPHA	4JPNA	4JPQA	4JPRA	4JQFA	4JQPA	4JQRA
4JQUB	4JR6A	4JR9A	4JRAC	4JRFA	4JS8A	4JTIA	4JTMA	4JV8B
4JVCA	4JW0A	4JWJA	4JX0A	4JX2A	4JXRA	4JXUA	4JYKA	4JZJC
4JZPA	4JZZA	4K0DA	4K0NA	4K12A	4K12B	4K1CA	4K1PA	4K2JA
4K47A	4K4KA	4K4OA	4K51A	4K5SA	4K6LG	4K6NA	4K7BA	4K7JA
4K82A	4K84A	4K8WA	4K90A	4K90B	4K92A	4K94C	4KBQC	4KBXA
4KDDA	4KDWA	4KEMA	4KF8A	4KFUA	4KG0A	4KGDA	4KGHA	4KGQC
4KH6A	4KH8A	4KH9A	4KHBA	4KIAA	4KJMA	4KK0A	4KK7A	4KKRA
4KKUA	4KKZA	4KL0A	4KM6A	4KMDA	4KOPA	4KP1A	4KP3C	4KPPA
4KQ7A	4KQ9A	4KQAA	4KQDA	4KQIA	4KQPA	4KQWA	4KRDB	4KRRA
4KRXA	4KSNA	4KT0C	4KT3A	4KT3B	4KT5C	4KT6A	4KT6B	4KTIA
4KTWA	4KU0A	4KU0D	4KUIA	4KV2A	4KV7A	4KV9A	4KVGB	4KW3A
4KWDA	4KWYA	4KYPA	4KZSA	4L0CA	4L0JA	4L0KA	4L0RA	4L0VA
4L1NA	4L2HA	4L2IA	4L2IB	4L2WA	4L3NA	4L3RA	4L3UA	4L4EA
4L4QA	4L57A	4L5GA	4L63A	4L68A	4L6UA	4L75A	4L7AA	4L7XA
4L8HA	4L8IA	4L8JA	4L8KA	4L9BA	4L9EA	4L9NA	4L9PA	4L9PB
4LA2A	4LANA	4LB0A	4LB8A	4LBAA	4LBUA	4LCTA	4LD1A	4LDAA
4LDVA	4LE3A	4LEBA	4LERA	4LF0A	4LFHD	4LFUA	4LG3A	4LGCA
4LGJA	4LH6A	4LHFA	4LHPA	4LHSA	4LIDA	4LJ0A	4LJ6A	4LJHA
4LJOA	4LJRA	4LJSA	4LKSA	4LL6A	4LL7A	4LLDA	4LLDB	4LLEA
4LLMA	4LLOA	4LLQA	4LM6A	4LMGA	4LMOA	4LMWA	4LMYA	4LN0C
4LNSA	4LO6B	4LONB	4LPIA	4LPQA	4LPSA	4LQ6A	4LQ8A	4LQEA
4LQKA	4LR4A	4LRJA	4LRTA	4LRTB	4LRVA	4LRZE	4LS9A	4LSCA
4LSDA	4LSWA	4LT5A	4LTBA	4LTTA	4LTYA	4LU2A	4LUAA	4LUMA
4LUNU	4LUPA	4LUQC	4LV5B	4LV8A	4LVFA	4LVIA	4LVNP	4LVPA
4LW8A	4LWUA	4LX2A	4LXRJ	4LY1A	4LYAA	4LZJA	4LZKA	4M0NA
4M0QA	4M0WA	4M1AA	4M1GA	4M1GH	4M1UA	4M1XA	4M2BA	4M3OA
4M3PA	4M51A	4M5BA	4M5DB	4M5EA	4M5RA	4M6TA	4M7RA	4M7TA
4M7XA	4M82A	4M83A	4M88A	4M8AA	4M8RA	4M91A	4M9VC	4MAAA
4MAKA	4MAXA	4MB0A	4MB7A	4MBOA	4MBYA	4MC3A	4MCDA	4MCJA
4MCOA	4MD5B	4MDAA	4MDWA	4MDYA	4ME2A	4ME3A	4ME7E	4MEAA
4MERA	4MESA	4MEWA	4MFIA	4MFKA	4MFUA	4MG3A	4MGQA	4MHLA
4MI7A	4MIJA	4MIWA	4MJ2A	4MJ7A	4MJFA	4MJGA	4MJKA	4MJSB
4ML1A	4MLDA	4MLMA	4MM2A	4MMOA	4MMSA	4MN4C	4MN5A	4MN9A
4MNCA	4MNNA	4MNOA	4MNRA	4MO0A	4MO1A	4MP8A	4MPCA	4MQDA

4MQWX	4MRTC	4MS4B	4MT2A	4MT4A	4MT8A	4MTEA	4MTHA	4MTMA
4MTUA	4MU3A	4MU6A	4MUBA	4MUQA	4MUVA	4MV4A	4MVEA	4MVTA
4MWIA	4MXNA	4MXTA	4MYVA	4MZ2A	4MZ7A	4MZAA	4MZCA	4MZGB
4MZJA	4MZVA	4MZYA	4N01A	4N04A	4N0HB	4N0HF	4N0NA	4N0RA
4N0TA	4N13A	4N1IA	4N1YA	4N2PA	4N2XA	4N30A	4N3PA	4N3SA
4N3YB	4N49A	4N4JA	4N4PA	4N5BA	4N6CA	4N6DA	4N6KA	4N6OA
4N6OB	4N6QA	4N74A	4N75A	4N77A	4N7QA	4N7RA	4N7RC	4N7WA
4N8NA	4N8PA	4N9WA	4N9XA	4NADA	4NAOA	4NB5A	4NBOA	4NBPA
4NBXA	4NC7A	4NCDA	4NCKA	4NCXA	4NDOA	4NDSA	4NE3A	4NE3B
4NEJA	4NESA	4NF0A	4NF1A	4NF7A	4NFAA	4NG0A	4NG2E	4NGDA
4NHBA	4NHEA	4NHRA	4NI6A	4NJ6A	4NK2A	4NK6A	4NKBA	4NKGB
4NKNA	4NKPA	4NKRA	4NL9A	4NLCA	4NLMA	4NLRA	4NM6A	4NMIA
4NMXA	4NMYA	4NN2A	4NN5A	4NN5B	4NNOA	4NNZA	4NOAA	4NOFA
4NOGA	4NOHA	4NONA	4NOOA	4NOOB	4NPDA	4NPLA	4NPTA	4NPUA
4NQ0A	4NQFA	4NQIA	4NQJA	4NQWB	4NRDA	4NRHB	4NS5A	4NSMA
4NSSA	4NSVA	4NT1A	4NTKA	4NTQA	4NTQB	4NUAA	4NUUA	4NUXA
4NV0A	4NV4A	4NWBA	4NWYA	4NXIA	4NXYA	4NYHA	4NYQA	4NZGA
4NZKA	4O06A	4O0CA	4O1IA	4O1WA	4O2HA	4O2TA	4O4FA	4O4OA
4O4YH	4O59O	4O5FA	4O5JA	4O5VA	4O65A	4O66A	4O6AA	4O6GA
4O6KA	4O6UA	4O6YA	4O7JA	4O87A	4O8QA	4O8SA	4O8UA	4O8VA
4O8WA	4O93A	4O93B	4O9BA	4O9DA	4OA3A	4OAGA	4OAYA	4OBMA
4OBOA	4OBXA	4OCIA	4OCVA	4OD6A	4ODKA	4OE8A	4OE8B	4OEBA
4OFKA	4OFZA	4OH7A	4OHCA	4OHJA	4OHUA	4OHXA	4OI3A	4OIEA
4OJ8A	4OJXA	4OKEA	4OKOA	4OKVE	4OKZA	4OL4A	4OLEA	4OLOA
4OLSA	4OLTA	4OM3A	4OM8A	4OMBA	4OMFB	4OMGA	4OMVA	4ON1A
4ONQA	4ONWA	4OOGA	4OOXA	4OOYA	4OPBA	4OPWA	4OQ1A	4OQ9A
4OQPA	4OQVA	4OR5A	4ORKA	4ORRA	4OTMA	4OTNA	4OTSA	4OU0A
4OU6A	4OU9A	4OUNA	4OUSA	4OV8A	4OVJA	4OVXA	4OVYA	4OW8A
4OWFA	4OWKA	4OWTA	4OX0A	4OXXA	4OY3A	4OY6A	4OYDB	4OZUA
4P04A	4P0TA	4P0ZA	4P17A	4P1MA	4P1NA	4P1ZA	4P2VA	4P32A
4P3AA	4P3FA	4P3HA	4P40A	4P49A	4P5EA	4P5NA	4P5XA	4P6BA
4P78A	4P78C	4P7AA	4P7OA	4P82A	4P9FA	4P9GA	4P9IA	4PAGA
4PD0A	4PD6A	4PDCE	4PDYA	4PE3A	4PE6A	4PEDA	4PEIA	4PERA
4PEUA	4PEVA	4PF3A	4PF4A	4PFSA	4PFYA	4PGRA	4PHJA	4PHQA
4PHZB	4PHZC	4PIOA	4PJ2A	4PJ2C	4PJRA	4PK9A	4PKEA	4PKFB
4PL0A	4PL3A	4PLZA	4PMOA	4PN6A	4PNOA	4PO6A	4PONA	4PP4A
4PP8C	4PPUA	4PQ0A	4PQQA	4PQZA	4PS6A	4PSFA	4PSRA	4PT7A
4PTSA	4PUIA	4PUXA	4PVCA	4PW0A	4PW2A	4PWNA	4PWWA	4PWYA
4PXCA	4PXUA	4PXYA	4PY9A	4PZ0A	4PZ3A	4PZ7A	4PZJA	4PZOA
4PZUA	4Q05A	4Q0PA	4Q1QA	4Q1ZA	4Q28A	4Q29A	4Q2LA	4Q2SA
4Q2UA	4Q2WA	4Q4FA	4Q4GX	4Q4W1	4Q4W2	4Q4W3	4Q4W4	4Q51A
4Q52A	4Q5GA	4Q5WA	4Q63A	4Q68A	4Q6JA	4Q6RA	4Q6TA	4Q6VA
4Q7IA	4Q7OA	4Q82A	4Q86A	4Q88A	4Q8GA	4Q98A	4Q9TA	4QA8A
4QAMB	4QASA	4QB0A	4QBBA	4QBDB	4QBLA	4QBUA	4QC6A	4QDCA

4QDGA	4QEKA	4QFLA	4QFTA	4QGOA	4QHQA	4QIKA	4QJBA	4QJFA
4QJKA	4QJVA	4QJVB	4QKDA	4QKYA	4QM6A	4QM9A	4QMAA	4QMFB
4QMHA	4QMKA	4QN8A	4QNDA	4QNUA	4QOSA	4QOZC	4QP5A	4QPMA
4QPNA	4QPWA	4QQ6A	4QQBX	4QQDA	4QQGA	4QRHA	4QRNA	4QRSB
4QSGA	4QTDA	4QTJA	4QTNA	4QU6A	4QUVA	4QVRA	4QWWC	4QXAB
4QXBA	4QXBB	4QXLA	4QY7A	4QYGA	4QYTA	4R12A	4R1BA	4R1DA
4R1DB	4R1JA	4R29A	4R2FA	4R2XA	4R2YA	4R33A	4R3NA	4R3OG
4R3QA	4R4KA	4R4XA	4R6HA	4R6IA	4R6UA	4R6YA	4R7RA	4R80A
4R9FA	4R9OA	4RAAA	4RAPA	4RBRA	4RCJA	4RDBA	4RDQA	4RE1C
4RECA	4REVA	4RFAA	4RG1A	4RGLA	4RGPA	4RGWA	4RGWB	4RHOA
4RHPA	4RHSA	4RHWE	4RJZA	4RK2A	4RK4A	4RK6A	4RKUG	4RKUH
4RKUK	4RKUN	4RL6A	4RMMA	4RO3A	4ROQA	4RP3A	4RPLA	4RU0A
4RU1A	4RUWA	4RV2A	4RWHA	4RWRA	4RWUA	4RXJA	4RXLA	4RXMA
4RXTA	4RXXA	4RY1A	4RY8A	4RYRA	4RZ0A	4S1AA	4S1HA	4S2VA
4TJVA	4TKBA	4TKXL	4TMDA	4TPNA	4TPSA	4TPSB	4TQ3A	4TR3A
4TR6A	4TRHA	4TRKA	4TSHA	4TT0A	4TTRA	4TV7A	4TVOA	4TVSA
4TVVA	4TW3A	4TW5A	4TWBA	4TWKA	4TXDA	4TXRA	4TY0A	4TYZA
4U0OB	4U12A	4U19A	4U1EG	4U1EI	4U1FA	4U3VA	4U4EA	4U4HA
4U5AA	4U5HA	4U5RA	4U5WA	4U6UA	4U6UB	4U77A	4U7IA	4U7UA
4U7UB	4U7UE	4U8FA	4U98A	4U9BA	4U9HL	4U9HS	4U9NA	4U9OA
4U9PA	4U9UA	4U9VB	4UA8A	4UABA	4UADE	4UAFB	4UC1A	4UE8A
4UF0A	4UMGA	4UMLA	4UN1B	4UNUA	4UOSA	4UP0A	4UQIA	4UQWA
4UQXA	4UR7A	4URJA	4US5A	4UT1A	4UTUA	4UUYA	4UUZC	4UV2A
4UVJA	4UVQA	4UW9A	4UWHA	4UXAA	4UXUA	4UY3A	4UYBA	4UYIA
4UZ1A	4UZ8A	4UZYB	4UZZA	4UZZB	4V03A	4V0KA	4V0PA	4V24A
4V28A	4V2BA	4V2YA	4V3DA	4V3IA	4W4KA	4W4KB	4W82A	4WANA
4WATA	4WD8A	4WE2A	4WJMA	4WPEA	4WW4B	4WWHA	4WY4A	4WY4B
4WY4C	4WY4D	4WZNA	4WZRA	4XUUA	4XVXA	5AHOA		

Table 1 Predicted MAE and accuracy at the threshold 25% of individual sequence on Manesh215 dataset

NO	Sequences	Length	Accuracy(25%)	MAE
1	119LA	162	0.808641975	0.076580305
2	153LA	185	0.854054054	0.085345503
3	1ABAA	87	0.827586207	0.088224812
4	1ABRB	267	0.74906367	0.102192408
5	1AFRA	345	0.831884058	0.087167959
6	1AFWA	238	0.840336134	0.073381442
7	1AMMA	174	0.752873563	0.097989876
8	1AMPA	291	0.886597938	0.063011656
9	1AOCA	175	0.691428571	0.128852238
10	1ATLA	200	0.83	0.074373501
11	1ATNA	371	0.784366577	0.102372199
12	1AXNA	323	0.857585139	0.06968745
13	1BBPA	173	0.832369942	0.075981451
14	1BDOA	80	0.775	0.111401505
15	1BEOA	98	0.826530612	0.083650351
16	1BFGA	126	0.793650794	0.083601375
17	1BGCA	121	0.826446281	0.085448862
18	1BHMB	208	0.783653846	0.09041616
19	1BIBA	117	0.803418803	0.089088789
20	1BKSB	392	0.87755102	0.061406818
21	1BMFG	44	0.772727273	0.085156032
22	1BNCA	159	0.830188679	0.082444656
23	1BTMA	251	0.876494024	0.070275623
24	1BTNA	106	0.867924528	0.075871099
25	1CEMA	363	0.790633609	0.080828774
26	1CEOA	94	0.861702128	0.073958885
27	1CEWI	108	0.824074074	0.087989244
28	1CFYA	133	0.84962406	0.069619219
29	1CHDA	198	0.787878788	0.081857999
30	1CHKA	238	0.903361345	0.070296487
31	1CHMA	401	0.890274314	0.064976392
32	1CMKE	348	0.781609195	0.091692126
33	1CNVA	283	0.844522968	0.076867153
34	1CSEE	274	0.835766423	0.073612074
35	1CSGA	120	0.758333333	0.098789233
36	1CSNA	293	0.822525597	0.087134384
37	1CYXA	158	0.841772152	0.086349859
38	1DEAA	266	0.827067669	0.083186767
39	1DELA	241	0.7593361	0.100807745
40	1DFJI	456	0.868421053	0.059109061

41	1DHRA	236	0.796610169	0.080494963
42	1DKTB	71	0.676056338	0.148492278
43	1DKZA	215	0.762790698	0.082356205
44	1DOSA	108	0.87037037	0.079527891
45	1DXYA	330	0.824242424	0.08863477
46	1ECEA	358	0.849162011	0.065841998
47	1ECPA	237	0.88185654	0.0700264
48	1EDEA	310	0.870967742	0.069536868
49	1EDGA	380	0.797368421	0.084996663
50	1EDTA	265	0.826415094	0.07479482
51	1ERVA	105	0.847619048	0.061399584
52	1ESCA	302	0.857615894	0.078697019
53	1EXNB	268	0.798507463	0.093165667
54	1EZMA	298	0.788590604	0.08879713
55	1FDSA	190	0.852631579	0.084392103
56	1FJMA	294	0.833333333	0.091337872
57	1FTPA	133	0.77443609	0.08818692
58	1FUAA	206	0.849514563	0.071445276
59	1GAIA	472	0.862288136	0.073194142
60	1GCBA	452	0.807522124	0.096789717
61	1GGGA	220	0.818181818	0.084415089
62	1GNDA	430	0.818604651	0.088375248
63	1GOTB	339	0.873156342	0.071372117
64	1GPCA	218	0.78440367	0.090883714
65	1GPLA	432	0.80787037	0.082048086
66	1GSAA	314	0.792993631	0.079116221
67	1GTMA	417	0.872901679	0.068568345
68	1HAVA	216	0.782407407	0.097530801
69	1HFCA	157	0.872611465	0.078926838
70	1HGXA	67	0.835820896	0.072674126
71	1HLBA	157	0.840764331	0.083951544
72	1HSBA	270	0.762962963	0.099387066
73	1HTPA	131	0.86259542	0.0859265
74	1IDAA	99	0.818181818	0.107012893
75	1IDOA	184	0.907608696	0.066977894
76	1IFCA	131	0.809160305	0.090525417
77	1IRKA	303	0.798679868	0.08629647
78	1ITGA	86	0.709302326	0.132354478
79	1JKWA	277	0.837545126	0.09216453
80	1KNBA	186	0.774193548	0.09728012
81	1KNYA	253	0.85770751	0.071007701
82	1KPTA	105	0.752380952	0.105444392
83	1KTEA	105	0.838095238	0.070825819

84	1KUHA	132	0.75	0.100226062
85	1LBAA	146	0.835616438	0.090664163
86	1LCLA	141	0.886524823	0.071915636
87	1LKIA	172	0.802325581	0.080226897
88	1LKKA	105	0.857142857	0.089792683
89	1LTSA	185	0.805405405	0.089826858
90	1M85A	475	0.749473684	0.115617065
91	1MAIA	119	0.924369748	0.068222474
92	1MAZA	27	0.740740741	0.124605548
93	1MBDA	153	0.882352941	0.070464031
94	1MKAA	171	0.789473684	0.099705803
95	1MLDA	313	0.881789137	0.064710054
96	1MMLA	251	0.796812749	0.099914478
97	1MOLA	94	0.808510638	0.114792605
98	1NARA	289	0.802768166	0.083655186
99	1NBAB	252	0.817460317	0.091174959
100	1NOXA	200	0.835	0.105219906
101	1NOZA	42	0.69047619	0.127120239
102	1OFGA	381	0.82152231	0.070419294
103	1ONRA	316	0.879746835	0.061795605
104	1OPRA	213	0.807511737	0.0904525
105	1OSPO	251	0.772908367	0.100969343
106	1PBCA	391	0.83887468	0.078651278
107	1PDAA	46	0.847826087	0.067106624
108	1PDOA	129	0.80620155	0.098212258
109	1PEAA	368	0.885869565	0.064454679
110	1PEXA	192	0.833333333	0.069936776
111	1PGSA	311	0.784565916	0.083134893
112	1PHEA	405	0.87654321	0.06223348
113	1PHPA	394	0.875634518	0.068406644
114	1PIOA	256	0.89453125	0.066915057
115	1PLCA	99	0.848484848	0.088497133
116	1PMIA	440	0.813636364	0.075269832
117	1PNEA	139	0.820143885	0.074929759
118	1POAA	118	0.771186441	0.098538351
119	1POCA	134	0.679104478	0.123444426
120	1POTA	322	0.860248447	0.063188529
121	1PPNA	212	0.830188679	0.080951347
122	1PUDA	372	0.814516129	0.078695972
123	1PYTA	94	0.776595745	0.111184141
124	1QAPA	289	0.809688581	0.085860336
125	1RA9A	159	0.836477987	0.085648233
126	1RCFA	169	0.869822485	0.069031764

127	1RECA	65	0.738461538	0.103482303
128	1RGSA	264	0.787878788	0.093506319
129	1RNLA	138	0.81884058	0.081670939
130	1RROA	108	0.787037037	0.077373847
131	1RSYA	3	0.784	0.096635231
132	1RVAA	244	0.827868852	0.088477823
133	1SBPA	309	0.854368932	0.077242339
134	1SFTB	380	0.889473684	0.063538574
135	1SIGA	55	0.690909091	0.11100767
136	1SLUA	81	0.75308642	0.113974345
137	1SMEA	329	0.811550152	0.087212999
138	1SMPI	100	0.76	0.099931316
139	1SRAA	151	0.834437086	0.095325259
140	1STDA	162	0.759259259	0.085562137
141	1STFI	98	0.724489796	0.103256636
142	1SVPA	160	0.78125	0.096212108
143	1TADC	318	0.830188679	0.085440693
144	1TFEA	142	0.85915493	0.099271974
145	1TFRA	77	0.753246753	0.07652052
146	1THVA	207	0.84057971	0.084451028
147	1THXA	108	0.787037037	0.080965605
148	1TIBA	269	0.847583643	0.07824502
149	1TMLA	286	0.807692308	0.082671979
150	1TUPC	195	0.784615385	0.099381995
151	1TYSA	263	0.817490494	0.092614898
152	1UBIA	76	0.815789474	0.107479806
153	1UBYA	348	0.813218391	0.082725945
154	1UDII	83	0.831325301	0.096911003
155	1UXYA	340	0.873529412	0.07172241
156	1VCAA	199	0.814070352	0.08869936
157	1VHHA	157	0.796178344	0.086575652
158	1VHRA	178	0.842696629	0.074495428
159	1VIDA	213	0.868544601	0.064606609
160	1VINA	252	0.865079365	0.072461441
161	1VLSA	146	0.808219178	0.091675392
162	1WBAA	171	0.789473684	0.095486597
163	1WHIA	122	0.803278689	0.117567572
164	1WHOA	94	0.808510638	0.082109916
165	1XFFA	238	0.852941176	0.080073254
166	1XGSA	295	0.850847458	0.075446038
167	1XNBA	185	0.837837838	0.08155408
168	1XVAA	292	0.797945205	0.086500884
169	1XYZA	320	0.859375	0.062981217

170	1YASA	256	0.85546875	0.07230153
171	1YSCA	421	0.857482185	0.075270693
172	1YTWA	283	0.798586572	0.104751497
173	256BA	106	0.858490566	0.055387874
174	2ABKA	211	0.834123223	0.094776372
175	2ARCA	161	0.850931677	0.081832137
176	2AYHA	214	0.817757009	0.081533189
177	2BBVC	12	0.833333333	0.091076509
178	2CBAA	258	0.829457364	0.076476187
179	2CCYA	127	0.818897638	0.092103281
180	2CHSA	114	0.807017544	0.092647415
181	2CTCA	307	0.876221498	0.071278732
182	2ENDA	137	0.817518248	0.101226121
183	2GDMA	153	0.888888889	0.066814317
184	2HFTA	86	0.825581395	0.092575658
185	2HHMA	266	0.906015038	0.057937625
186	2HPDA	457	0.866520788	0.072121586
187	2I1BA	153	0.810457516	0.087497194
188	2LIVA	344	0.875	0.061894069
189	2MTAC	147	0.734693878	0.10715178
190	2NACA	374	0.836898396	0.083134821
191	2PGDA	473	0.837209302	0.071477752
192	2PHLA	200	0.82	0.091987559
193	2PHYA	125	0.856	0.071169851
194	2PIAA	321	0.803738318	0.0821447
195	2PSPA	105	0.714285714	0.11423624
196	2RN2A	155	0.851612903	0.083124027
197	2RSPB	58	0.74137931	0.107621963
198	2SCPA	174	0.816091954	0.081236441
199	2SILA	381	0.82152231	0.088508922
200	2SNSA	141	0.893617021	0.08211819
201	2TDXA	139	0.841726619	0.079689545
202	2TYSA	178	0.887640449	0.066620635
203	3CHYA	128	0.8359375	0.077262099
204	3COXA	429	0.801864802	0.081565145
205	3GRSA	461	0.793926247	0.106481556
206	3MDDA	385	0.841558442	0.089780529
207	3MINB	522	0.894636015	0.054535955
208	3NLLA	138	0.884057971	0.06812749
209	3SDHA	145	0.84137931	0.080691576
210	5P21A	166	0.801204819	0.084098368
211	5PTPA	222	0.851351351	0.082013829
212	6GSVA	217	0.852534562	0.066639379

213	6PFKA	319	0.833855799	0.073301908
214	7RSAA	124	0.758064516	0.100567933
215	8ATCB	146	0.828767123	0.098722845
